# A Systematic Review of Obesity and Binge Eating Associated Impairment of the Cognitive Inhibition System

**DOI:** 10.3389/fnut.2021.609012

**Published:** 2021-04-29

**Authors:** Elodie Saruco, Burkhard Pleger

**Affiliations:** Department of Neurology, BG University Clinic Bergmannsheil, Ruhr-University Bochum, Bochum, Germany

**Keywords:** obesity, impulsivity, central nervous system, inhibition, functional magnetic resonance imaging, binge eating disorder

## Abstract

Altered functioning of the inhibition system and the resulting higher impulsivity are known to play a major role in overeating. Considering the great impact of disinhibited eating behavior on obesity onset and maintenance, this systematic review of the literature aims at identifying to what extent the brain inhibitory networks are impaired in individuals with obesity. It also aims at examining whether the presence of binge eating disorder leads to similar although steeper neural deterioration. We identified 12 studies that specifically assessed impulsivity during neuroimaging. We found a significant alteration of neural circuits primarily involving the frontal and limbic regions. Functional activity results show BMI-dependent hypoactivity of frontal regions during cognitive inhibition and either increased or decreased patterns of activity in several other brain regions, according to their respective role in inhibition processes. The presence of binge eating disorder results in further aggravation of those neural alterations. Connectivity results mainly report strengthened connectivity patterns across frontal, parietal, and limbic networks. Neuroimaging studies suggest significant impairment of various neural circuits involved in inhibition processes in individuals with obesity. The elaboration of accurate therapeutic neurocognitive interventions, however, requires further investigations, for a deeper identification and understanding of obesity-related alterations of the inhibition brain system.

## Introduction

Over the last decade, health care professionals have been exposed to the emergence of a new form of addiction—food addiction (1). Eating has always been a basic human behavior primarily devoted to the maintenance of homeostasis. Nonetheless, high-energy food products (i.e., rich in fat and/or sugar), which are everyday more available on the market, are engaging a strong reward response (2–4) and, due to those strong reinforcing properties (5), lead to addictive behaviors, like craving, similar to those caused by drugs (6). Craving, or a strong desire to consume a product, is associated with high sensitivity of the reward system (7). This hypersensitivity of the reward system has been well-documented in individuals with obesity (8–12). Notably, in response to the mere visualization of food items, neuroimaging studies report excessive activations of several brain regions strongly involved in food intake and reward processes [e.g., (9, 13)], hence fostering the excessive desire to eat (14).

Parallel to reward system hyperactivity, but less extensively investigated, a hypoactivity of the inhibition system was observed in individuals with obesity. Specifically, (f)MRI studies reported decreased gray matter volume (15) and functional activity of the frontal cortex (16), the brain region highly responsible for inhibition processes (17, 18). It has further been discovered that the repeated exposure to high-energy food will lead to a decrease of the availability of dopaminergic D2 receptors in the striatum (19). Not without consequence, this dopaminergic alteration directly impairs prefrontal activity (20, 21) and, thus, associated inhibitory control (22). Consequently, in individuals with obesity, a hypoactive inhibitory system will inevitably fail to counteract reward system hyperfunctioning (19).

Such imbalance between the two systems will behaviorally translate into an impulsive way to act (23), characterized by difficulties to override eating temptation (24) and lead to obesity. A strong association between body mass index (BMI) and impulsivity has been established in several studies (25–27). Impulsive behavior can result into poor response inhibition ability (28), which, interestingly, was found to correlate positively with overeating (29) and BMI (30, 31). Basically, those studies reported poor performances during go/no-go and stop signal tasks, with an impaired ability to inhibit from responding during no-go and stop trials. Impulsivity can also result from poor cognitive ability to override temptation. Several behavioral studies reported such insufficiently suppressed temptations in obese individuals using delay discounting, where participants have to choose between immediate or bigger although delayed reward, or craving regulation tasks, where participants are asked to use mental strategies to refrain from eating desire (32–34).

Interestingly, the presence of binge eating disorder (BED) leads to even greater impulsive traits (35, 36). This disorder, characterized by recurring short periods of uncontrolled consumption of abnormally large quantities of food, affects at least 25% of the obese population (37). Although overeating may not be considered as dangerous as drug consumption, this behavior leads to obesity with all its comorbidities (except in the case of purge, i.e., bulimia, not considered in this review since it does not lead to obesity-related devastating consequences on various aspects of health).

Recent brain stimulation studies provided promising results on the control of food craving and food intake (38). Specifically, studies showed that modulating the dorsolateral prefrontal cortex (39, 40), nucleus accumbens (41), and hypothalamic area (42) substantially reduced food craving. However, inhibitory control does not exclusively rely on the functioning of those three brain regions. It rather involves a complex neural network in which key regions and their way of communication remain largely unexplored.

Considering the importance of a precise identification of the different brain areas involved in the functioning of inhibition processes for the elaboration of relevant and accurate neurocognitive therapies, we propose in this review to gather the results provided by neuroimaging studies. The main aims are to [1] provide a detailed report of the brain regions showing obesity-related impaired function during response inhibition and [2] examine whether BED leads to similar although steeper neural deterioration.

## Methods

To identify studies aiming at investigating the neural basis of the inhibitory system in obese individuals, we used Google Scholar and PubMed databases using the following keywords: impulsive, impulsivity, inhibition, inhibitory system, and executive functions, each combined with the following terms: food, feeding behavior, obesity, and obese. Additional papers were also found from the reference lists of the selected papers. To comply with inclusion, studies had to [1] use a behavioral task specifically designed for the assessment of impulsivity, which [2] participants completed during functional magnetic resonance imaging (MRI) or magnetoencephalography (MEG), and [3] entail a lean control group (except in the specific case of comparison between obese with and without binge eating disorder). Moreover, participants from the experimental group had to [4] be obese (BMI ≥ 30) and not “only” overweight (25 < BMI < 29). Nonetheless, due to the lack of studies investigating impulsivity in individuals with BED, we decided to also include studies where participants were normal-weight binge eaters. Brain activation data were extracted from the MNI coordinates reported in the results sections of the original articles.

## Results

### Study Selection

**Figure 1:**
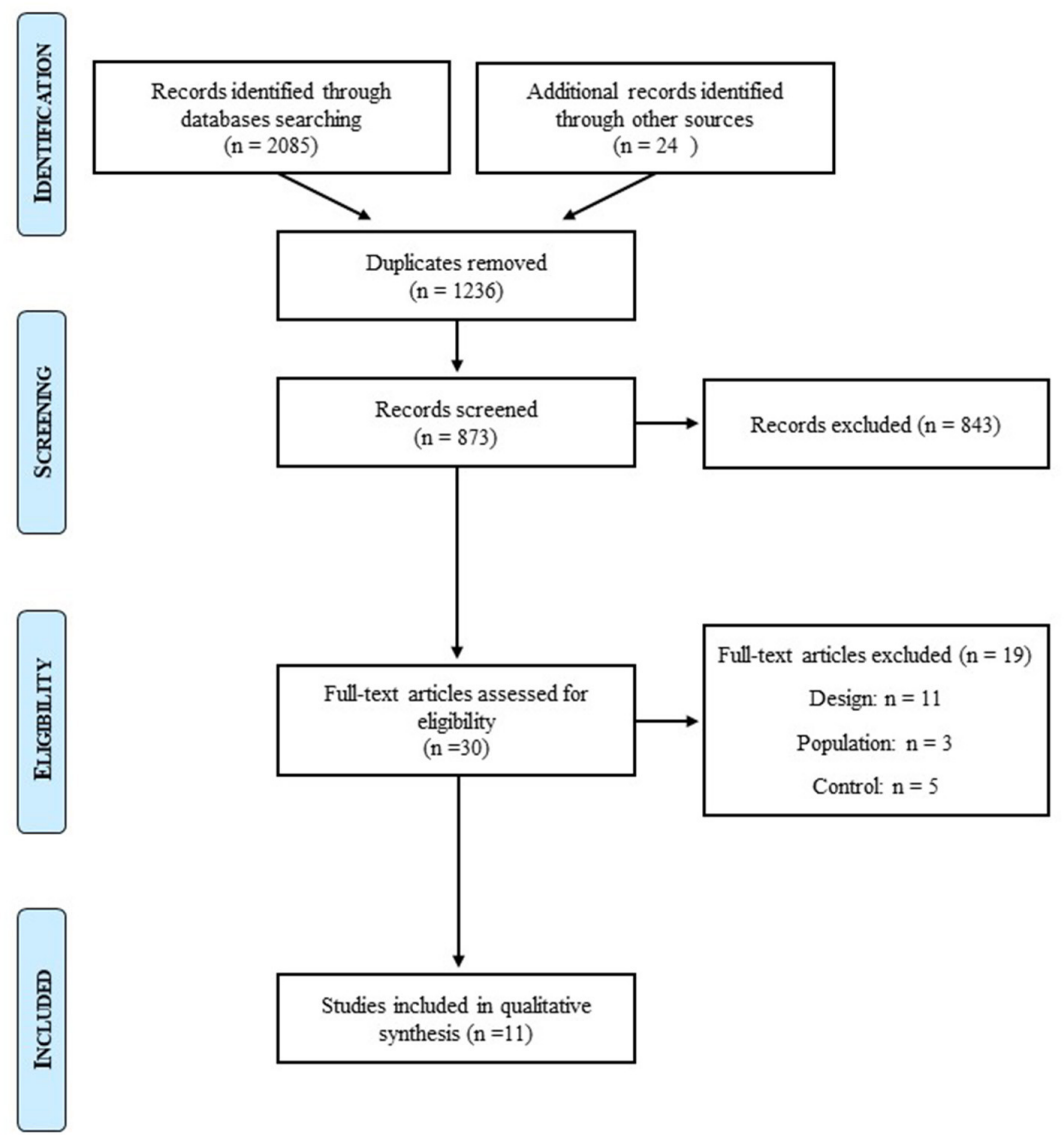
Prisma flow diagram.

The initial search identified 3,009 studies. After removal of duplicates and exclusions through title and abstract screening, 30 studies were assessed for eligibility. For design, population, and control reasons, 19 studies were excluded. After we further searched for articles on lean individuals with BED, one more article was selected. Twelve studies were hence included in this systematic review of the literature (Figure 1).

### Functional Activity

**Figure 2:**
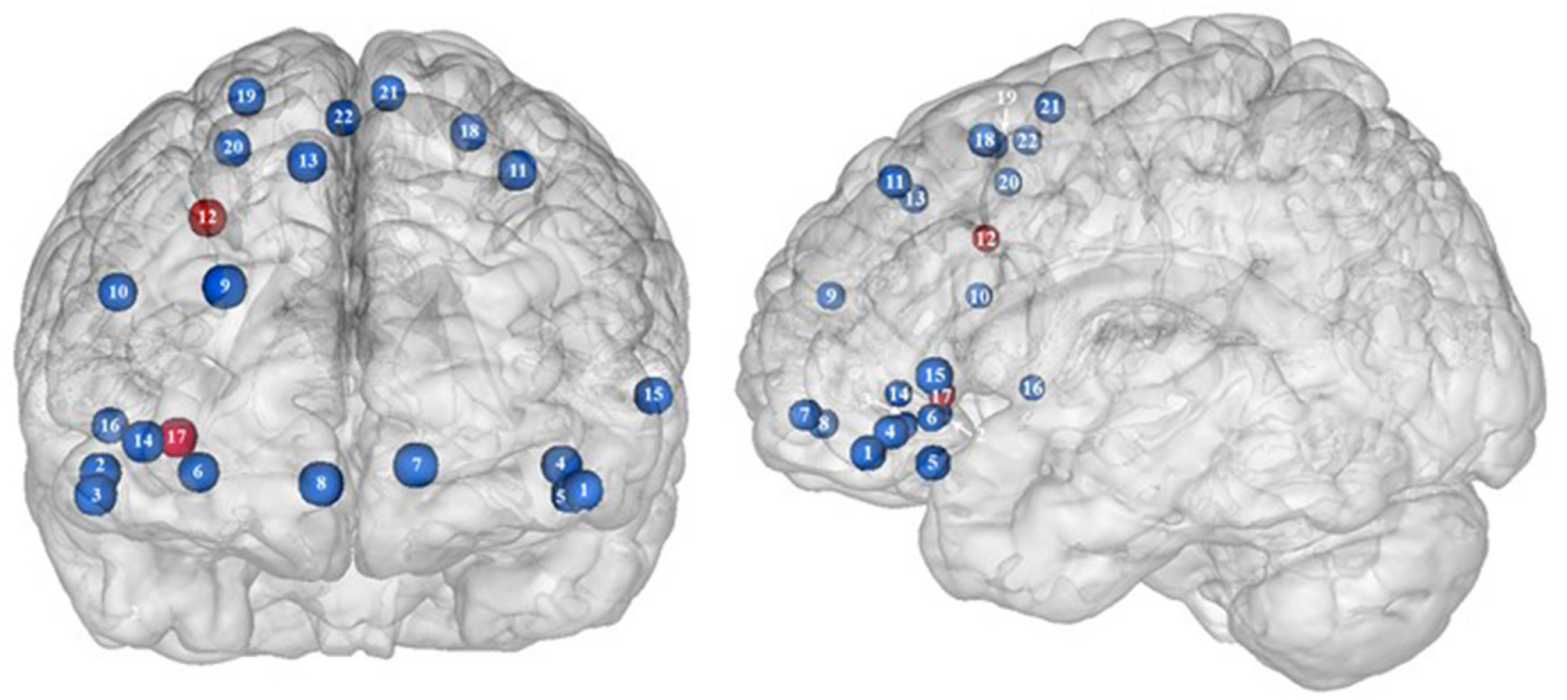
Functional activity in the frontal regions during inhibition. Blue and red colors, respectively, indicate hypo- and hyperactivity, respectively, in obese in comparison with lean participants during inhibition (see Table 2 for precise coordinates). 1–3 = orbitofrontal cortex (43), 4–5 = orbitofrontal cortex (48), 6 = orbitofrontal cortex (53), 7–8 = ventromedial prefrontal cortex (43), 9 = dorsomedial prefrontal cortex (48), 10 = dorsolateral prefrontal cortex (53), 11 = dorsolateral prefrontal cortex (50), 12 = dorsolateral prefrontal cortex (52), 13 = dorsolateral prefrontal cortex (43), 14 = ventrolateral prefrontal cortex (43), 15–16 = ventrolateral prefrontal cortex (48), 17 = ventrolateral prefrontal cortex (49), 18–20 = premotor cortex (43), 21–22 = premotor cortex (48).

**Figure 3:**
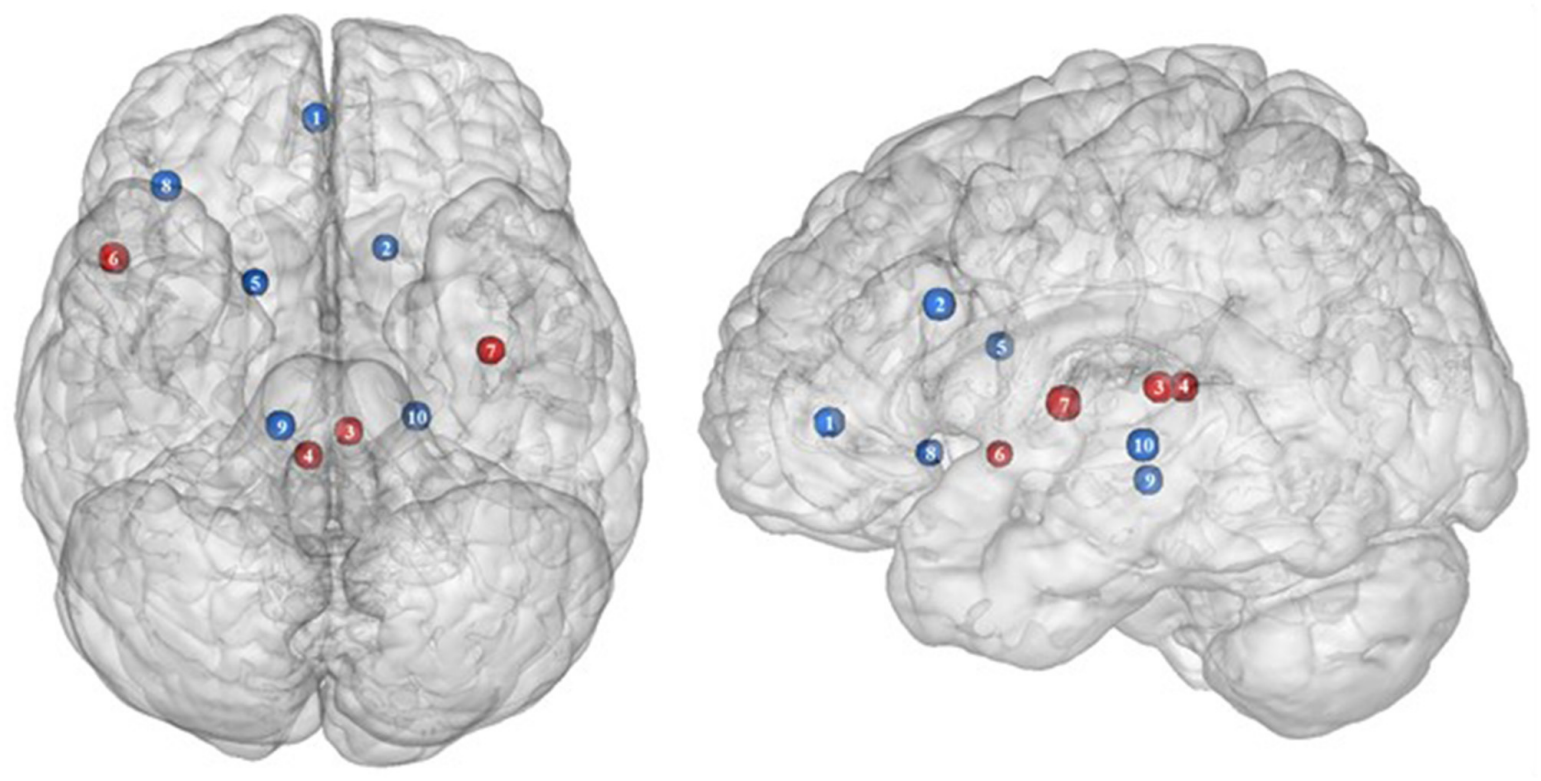
Functional activity in the limbic regions during inhibition. Blue and red colors indicate hypo- and hyperactivity, respectively, in obese in comparison with lean participants during inhibition (see Table 3 for precise coordinates). 1 = anterior cingulate cortex (46), 2 = anterior cingulate cortex (53), 3–4 = posterior cingulate cortex (53), 5 = dorsal caudate nuclei (53), 6 = insula (43), 7 = insula (32), 8 = insula (32), 9 = parahippocampal gyrus (49), 10 = thalamus (49).

In the 12 identified studies that met the inclusion criteria (Table 1), 10 reported different patterns of activation during response inhibition in obese in comparison with lean individuals (*n* = 8) or in obese with BED in comparison with obese without BED (*n* = 2) and one in lean individuals with BED in comparison with lean without BED. Differences were mainly observed in the frontal (Table 2, Figure 2) and limbic regions (Table 3, Figure 3), but also in the visual and inferior parietal cortices and the Rolandic operculum (Table 4, Figure 3). One study showed an inverted U-shaped pattern of activation in the insula, claustrum, and putamen (Table 5), hence reporting disparate neural activity during inhibition depending on obesity severity (32). Hypoactivity in the frontal and visual cortices reported in obese individuals during inhibition, but also in the temporal cortex, was found to be exacerbated in obese individuals with BED (Table 6). Only Carbine et al. (45) found no difference between their obese and lean participants' neural activity during inhibition.

**Table 1:** Neuroimaging studies assessing inhibition in obese in comparison with lean individuals.

**References**	**Participants**	**BMI**	**Task**	**Imaging technique**
Batterink et al. (43)	*n* = 29 (All W; M_age_ = 15.7 ± 0.93)	Range = 17.3–38.9	Food-specific go/no-go	fMRI
Balodis et al. (44)	*n* = 35 (W = 19; Obese with BED: M_age_ = 47.6 ± 12.7; Obese without BED: M_age_ = 35.4 ± 9.3; Lean: M_age_ = 32.7 ± 11.3)	Obese without BED: M = 34.6 ± 4.1; Obese with BED: M = 37.1 ± 3.9; Lean: M = 23.2 ± 1.1	General stroop color-word interference	fMRI
Carbine et al. (45)	*n* = 54 (50% W; M_age_ = 24.65 ± 7.31)	BMI > 30 *n* = 19; 25 < BMI < 30 *n* = 18; BMI < 25 *n* = 17;	Food-specific go/no-go	fMRI
Dietrich et al. (32)	*n* = 43 (All W; M_age_ = 26.7 ± 3.5)	Range = 19.4–38.8; M = 27.5 ± 5.3	Food-specific admit/regulate craving	fMRI
He et al. (46)	*n* = 30 (W = 17; M_age_ = 19.7 ± 1.7)	Range = 19.1–33.7; M = 23.1 ± 3.0	Food-specific go/no-go	fMRI
Hege et al. (47)	*n* = 34 All W; Obese with BED: *n* = 17, M_age_ = 41.88 ± 8.46; Obese without BED: *n* = 17, M_age_ = 41.35 ± 12.33	Obese with BED: M = 34.01 ± 5.58; Obese without BED: M = 36.52 ± 4.89	General go/no-go	MEG
Hendrick et al. (48)	*n* = 43 (All W; Obese: n = 13, M_age_ = 34.8 ± 9.6; Intermediate: *n* = 12, M_age_ = 33.2 ± 16.7; Lean: n = 18, M_age_ = 26.2 ± 6.7)	Obese: BMI > 30; Intermediate: 22 < BMI < 30; Lean: BMI < 22	General stop signal	fMRI
Hsu et al. (49)	*n* = 40 (All W; Obese: *n* = 20; Lean: *n* = 20)	Obese: BMI > 27; Lean: BMI < 24	General go/no-go	fMRI
Janssen et al. (50)	*n* = 76 (W = 65; M_age_ = 31.5 ± 10.7)	Range = 19–35; M = 26.4 ± 3.8	Food-specific stroop color-word interference	fMRI
Oliva et al. (51)	*n* = 42 (Lean with BED: *n* = 21, W = 17, M_age_ = 23.9 ± 3.19; Lean without BED: *n* = 21; W = 16; M_age_ = 25.23 ± 3.08)	Lean with BED: M = 22.3 ± 2.1; Lean without BED: M = 21.29 ± 2.02	General and Food-specific go/no-go and stop signal	fMRI
Scharmüller et al. (52)	*n* = 26 (All W; Obese: n = 14, M_age_ = 26.6 ± 4.5; Lean: 25.6 ± 6.7)	Obese: M = 31.5 ± 5.2; Lean: M = 20.6 ± 1.3	Food-specific admit/regulate craving	fMRI
Tuulari et al. (53)	*n* = 41 (All W; Obese: *n* = 27, M_age_ = 42.1 ± 9.3; Lean: *n* = 14, M_age_ = 44.9 ± 11.9)	Obese: M = 41.4 ± 3.9; Lean: M = 22.6 ± 2.7	Food-specific admit/regulate craving	fMRI

*BED, binge eating disorder; BMI, body mass index; fMRI, functional magnetic resonance imaging; M, mean; M_age_, mean age (in years ± standard deviation); MEG, magnetoencephalography; W, women*.

**Table 2:** Neural activity in frontal regions during inhibition in obese in comparison with lean individuals.

	**BA**	**Hemisphere**	**Coordinates**			**Results**
			***x***	***y***	***z***	
**PREFRONTAL CORTEX**						
**Orbitofrontal**						
Batterink et al. (43)	47	L	−39	33	−9	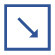
	47	R	45	33	−6	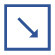
	47	R	45	42	−9	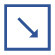
Hendrick et al. (48)	47	L	−36	29	−5	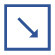
	47	L	−39	20	−11	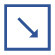
Tuulari et al. (53)	47	R	28	32	−6	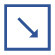
**Medial**						
*vmPFC*						
Batterink et al. (43)	10	L	−9	54	−3	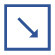
	10	R	6	54	−6	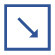
*dmPFC*						
Hendrick et al. (48)	10	R	21	56	25	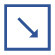
**Lateral**						
*dlPFC*						
Tuulari et al. (53)	9	R	44	22	26	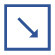
Janssen et al. (50)	8	L	−28	32	50	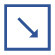
Scharmüller et al. (52)	8	R	28	18	40	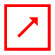
Batterink et al. (43)	8	R	9	33	48	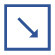
*vlPFC*						
Batterink et al. (43)	46	R	36	42	0	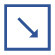
Hendrick et al. (48)	45	L	−57	17	7	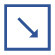
	44	R	48	8	1	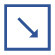
Hsu et al. (49)	45	R	32	30	0	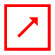
**PREMOTOR CORTEX**						
Batterink et al. (43)	6	L	−21	12	57	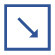
	6	R	21	15	63	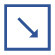
	6	R	24	12	54	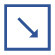
Hendrick et al. (48)	6	L	−6	−1	67	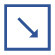
	6	R	3	5	61	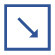

*Blue arrows indicate hypoactivity in obese in comparison with lean participants. Red arrows indicate hyperactivity in obese in comparison with lean participants*.

*BA, Brodmann area; dlPFC, dorsolateral prefrontal cortex; dmPFC, dorsomedial prefrontal cortex; vlPFC, ventrolateral prefrontal cortex; vmPFC, ventromedial prefrontal cortex*.

**Table 3:** Neural activity in limbic regions during inhibition in obese in comparison with lean individuals.

	**BA**	**Hemisphere**	**Coordinates**			**Results**
			***x***	***y***	***z***	
**CINGULATE CORTEX**						
**Anterior**						
He et al. (46)	32	R	4	44	4	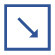
Tuulari et al. (53)	24	L	−14	16	30	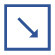
**Posterior**						
Tuulari et al. (53)	29	L	−4	−32	12	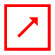
	29	R	6	−38	12	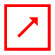
**DORSAL CAUDATE NUCLEI**						
Tuulari et al. (53)	48	R	20	6	22	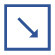
**Insula**						
Batterink et al. (43)	13	R	51	9	−6	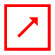
Dietrich et al. (32)	13	L	−39	−12	9	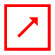
Hendrick et al. (48)	13	R	39	26	−5	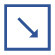
**PARAHIPPOCAMPAL GYRUS**						
Hsu et al. (49)	36	R	12	−30	−10	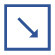
**Thalamus**						
Hsu et al. (49)	50	L	−20	−28	0	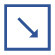

*Blue arrows indicate hypoactivity in obese in comparison with lean subjects. Red arrows indicate hyperactivity in obese in comparison with lean subjects*.

*BA, Brodmann area*.

**Table 4:** Other brain regions showing different patterns of activity during inhibition in obese in comparison with lean individuals.

	**BA**	**Hemisphere**	**Coordinates**			**Results**
			***x***	***y***	***z***	
**Visual cortex**						
Hendrick et al. (48)	17	L	−18	−70	4	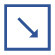
	17	L	−15	−76	16	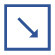
	17	R	9	−94	−2	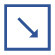
	17	R	12	−94	10	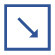
	18	R	21	−64	16	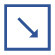
**Rolandic operculum**						
Hsu et al. (49)	1	R	58	−8	14	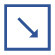
	1	R	38	−16	18	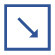
**Inferior parietal cortex**						
Hendrick et al. (48)	40	L	−60	−37	34	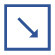
	40	R	60	−40	46	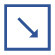

*Blue arrows indicate hypoactivity in obese in comparison with lean participants. Red arrows indicate hyperactivity in obese in comparison with lean participants*.

*BA, Brodmann area*.

**Table 5:** Regions showing an inverted U-shaped pattern of activity during inhibition in obese in comparison with lean individuals.

	**BA**	**Hemisphere**	**Coordinates**			**Results**
			***x***	***y***	***z***	
**Insula**						
Dietrich et al. (32)	13	L	−39	−12	9	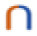
**Claustrum**						
Dietrich et al. (32)		L	−30	−3	−18	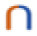
**Putamen**						
Dietrich et al. (32)	49	L	−33	−9	−3	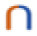

*U-inverted symbols indicate increased neural activity in class I obese (BMI = 30) in comparison with lean participants, followed by a decrease for obesity classes II and III (BMI = 35–40), reaching similar levels of activation as lean participants*.

*BA, Brodmann area*.

**Table 6:** Regions showing different patterns of activity during inhibition in obese individuals with binge eating disorder in comparison with individuals without.

		**BA**	**Hemisphere**	**Coordinates**			**Results**
				***x***	***y***	***z***	
**PREFRONTAL CORTEX**							
**Medial**							
*vmPFC*							
Oliva et al. (51)	*	10	R	43	53	2	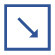
	*	10	R	33	56	2	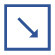
*dmPFC*							
Oliva et al. (51)	****	10	L	−31	49	26	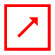
**Lateral**							
*dlPFC*							
Hege et al. (47)		9	R	44	30	28	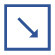
Oliva et al. (51)	****	46	L	−45	42	14	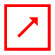
*vlPFC*							
Balodis et al. (44)		46	L	−30	36	9	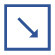
**VISUAL CORTEX**							
Balodis et al. (44)		19	L	−42	−87	9	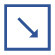
		19	R	45	−87	15	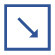
Oliva et al. (51)	*	18	R	36	−88	−6	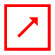
	**	18	R	36	−88	−6	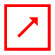
**SENSORIMOTOR CORTEX**							
**Primary motor cortex**							
Oliva et al. (51)	**	4	L	−38	−21	58	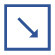
**Premotor cortex**							
Oliva et al. (51)	***	6	R	15	−25	46	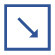
	***	6	R	22	−25	42	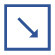
**Primary sensory cortex**							
Oliva et al. (51)	***	1	R	36	−28	46	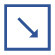
**CEREBELLUM**							
Oliva et al. (51)	*		L	−34	−84	−38	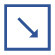
	*		L	−45	−67	−22	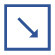
	**		L	−45	−67	−22	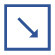
	**		L	−24	−81	−26	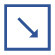
	**		L	−13	−70	−46	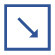
	**		L	−13	−39	−38	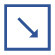
	***		L	−17	−74	−22	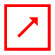
**PRECUNEUS**							
Oliva et al. (51)	*	7	R	1	−63	42	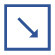
	*	7	R	5	−67	54	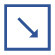
	**	7	R	5	−70	50	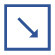
	**	7	R	5	−63	42	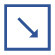
**PUTAMEN**							
Oliva et al. (51)	**	49	R	26	14	−2	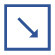
	**	49	R	12	7	−2	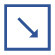
**TEMPORAL GYRUS**							
Balodis et al. (44)		37	R	60	−63	−12	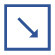

*Blue arrows indicate hypoactivity in obese individuals with binge eating disorder in comparison with those without binge eating disorder. Red arrows indicate hyperactivity in individuals with binge eating disorder in comparison with those without binge eating disorder*.

*BA, Brodmann area; dlPFC, dorsolateral prefrontal cortex; dmPFC, dorsomedial prefrontal cortex; vlPFC, ventrolateral prefrontal cortex; vmPFC, ventromedial prefrontal cortex*.

* = general go/no-go, ** = food-specific go/no-go, *** = general stop signal, **** = food-specific stop signal.

### Functional Connectivity

Two from the nine identified studies assessed functional connectivity during inhibition and reported either strengthened or U-shaped patterns of connectivity in obese in comparison with lean individuals (Table 7).

**Table 7:** Neural connectivity during inhibition in obese in comparison with lean individuals.

	**BA**	**Hemisphere**	**Coordinates**			**Results**
			***x***	***y***	***z***	
**FRONTAL CORTEX**						
Tuulari et al. (53)						
**Seed: dlPFC**	8	L	−42	14	42	
Putamen	49	R	32	−20	4	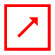
Cingulate cortex	23	R	30	−64	6	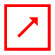
SMA	6	R	8	−8	64	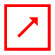
**Seed: Pre-SMA**	8	R	4	25	38	
Precuneus	31	L	−8	−54	36	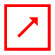
Cingulate cortex	32	R	10	16	36	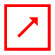
Inferior parietal cortex	39	R	42	−66	48	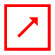
	39	R	52	−56	46	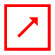
**PARIETAL CORTEX**						
Tuulari et al. (53)						
**Seed: precuneus**	7	R	11	−72	58	
SMA	6	R	10	−20	66	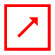
vlPFC	44	R	48	12	12	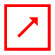
Sensory cortex	1	L	−20	−30	68	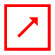
**BASAL GANGLIA**						
Dietrich et al. (32)						
**Seed: putamen**	49	L	−33	−9	−3	
dlPFC	9	L	−24	33	30	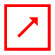
	9	L	−33	27	24	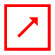
	8	L	−12	21	45	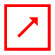
	8	R	9	42	45	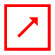
	8	L	−15	33	48	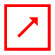
dmPFC	10	L	−33	45	30	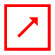
**Seed: amygdala**		L	−30	−3	−18	
Pallidum	51	L	−15	0	0	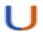
Putamen	49	L	−21	18	3	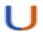
Visual cortex	17	L	−3	−90	6	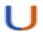
	18	L	−6	−81	−9	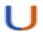

*Red arrows indicate strengthened connectivity in obese in comparison with lean participants. U-shaped symbols indicate weaker neural connectivity in class I obese (BMI = 30) in comparison with lean participants, but stronger connectivity for obesity classes II and III (BMI = 35–40), reaching similar connection strengths as lean participants*.

*BA, Brodmann area; dlPFC, dorsolateral prefrontal cortex; dmPFC, dorsomedial prefrontal cortex; pre-SMA, presupplementary area; SMA, supplementary area; vlPFC, ventrolateral prefrontal cortex*.

## Discussion

The studies included in this review used go/no-go, stop signal, Stroop, and craving control tasks to investigate inhibition processes in individuals with obesity. Designed to assess response inhibition, the go/no-go (action restraint) and stop signal tasks (action cancellation) engage overlapping but also different brain activations during successful inhibition. More precisely, while the medial prefrontal cortex and the insula were found to be engaged during both tasks, the go/no-go task triggers further activation in the fronto-parietal network and the stop signal task in the cingulo-opercular network (54). The Stroop task, assessing inhibition of prepotent response tendencies, has been shown to mainly recruit the prefrontal, anterior cingulate, and posterior parietal cortices (55–57). Despite stronger cognitive engagement required during craving control tasks, the intentional regulation of food desire and response inhibition share mainly similar networks. Specifically, in comparison with passively viewed food items or allowed food craving, regulation of food craving was also found to engage a large brain network encompassing the insula, prefrontal cortex, temporal parietal junction, and the supplementary motor area (58). Here, we review to what extent those neural networks are impaired in individuals with obesity. We provide a list of brain regions together with their precise anatomical locations and discuss their potential functions during inhibition processes.

### Frontal Regions

Frontal regions, including the prefrontal (PFC) and premotor cortices, have been consistently found to be involved in the inhibitory system (59–62). This role endorsed by the frontal regions in inhibitory processes also applies to the specific context of eating behavior (58). The results of this review of the literature confirm the major involvement of frontal regions during eating control in the obese population. However, over the 10 studies we identified that directly compared brain activity between lean and obese participants during an inhibition task, seven of them clearly reported an altered functioning of frontal regions, mostly characterized by hypoactivity (Figure 2).

#### Prefrontal Cortex

Compelling data from the studies that investigated the neural correlates of behavioral inhibition in obese in comparison with lean individuals clearly emphasize a hypoactivity of the PFC in the totality of both its medial and dorsal parts, suggesting obesity to be linked to a global impairment of this brain region, known to be a central hub for inhibitory processes.

##### Orbitofrontal Cortex

Located in the inferior part of the frontal lobe, the orbitofrontal cortex (OFC), known to be involved in encoding reward value and decision-making (63), plays a major role in the value attributed to food and subsequent eating behavior. More precisely, the lateral OFC encodes the objective nutritive value of food and integrates it within the medial OFC, which will in turn attribute the subjective value of the food item (64). Moreover, the OFC is a key node of the cognitive and, more specifically, emotion inhibition system (65). Interestingly, a decrease of the OFC metabolism has been observed in cocaine and alcohol abusers, probably on the grounds of decreased sensitivity to inhibitory (GABA) neurotransmission (20). Therefore, results from Batterink et al. (43), Hendrick et al. (48), and Tuulari et al. (53), reporting decreased activation of the OFC among obese participants during response inhibition specific to food, may suggest an alteration of the food value perception and/or emotion inhibition function(s) due to the addictive dimension of eating behavior in obese individuals.

##### Medial Prefrontal Cortex

The anterior part of the PFC (BA10) plays a central role in cognitive processes. BA10 is parcellated into an inferior and a superior part, respectively, named ventromedial and dorsomedial PFC. While each of these subregions endorses distinct cognitive roles, such as emotional regulation, salience attribution (66), and food valuation process (67) for the ventromedial PFC and *inter alia* decision-making (68–70) and uncertainty processing (71) for the dorsomedial PFC, both have been shown to play a role in inhibition processes. For instance, decreased gray matter volumes of the dorsomedial (72, 73) and lesions of the ventromedial PFC (74, 75) were found to be linked to increased levels of impulsivity. As previously observed among addicted gambler and heavy smokers (76, 77), this review of the literature revealed that obese individuals also show a hypofunctioning of both medial PFC regions during inhibition (43, 48).

##### Lateral Prefrontal Cortex

The lateral part of the PFC entails the dorsolateral and ventrolateral PFC. Postulated to be involved in cognitive inhibition processes (78, 79), the dorsolateral PFC plays an important role in the regulation of food craving (80, 81). Essential to the downregulation of high-energy food reward, this region has been shown to be critical for dietary self-control (82). Thus, considered as a key node of eating behavior control, the dorsolateral PFC has been the subject of numerous brain stimulation studies, showing that increased activation of this region allowed an improvement of resistance to food stimuli (38). In the same vein, higher levels of activity in the dorsolateral PFC were shown to be a good predictor of diet success in obese individuals (83, 84). Surprisingly, when comparing the activity of this region between obese and lean participants, only three studies found a significant difference in activations during food-specific inhibition, characterized by a hypofunctioning BA8 (43, 50) and BA9 (53). Scharmüller et al. (52), however, reported the opposite pattern of activation in BA8 (i.e., hyperactivity in comparison with lean participants). Interestingly, beyond its implication in inhibition (85), BA8 was found to be involved in uncertainty, with increased activations being correlated to the degree of uncertainty (86, 87). This could mean that the conviction driving eating refrainment may be more unstable in obese individuals. Together, results collected from dorsolateral PFC could reflect a food-specific impairment in both response inhibition and decision-making processes.

Decreased patterns of activity were also reported in parts of both the left [BA45, (48)] and right ventrolateral PFC [BA46, (43); BA44, (48)]. The ventrolateral PFC was also shown to be involved in inhibitory processes (88) and, more particularly, to be a critical substrate of dietary self-control (89). The results from Batterink et al. (43) and Hendrick et al. (48) corroborate those from Tuulari et al. (53) observed in the dorsolateral PFC, hence extending hypofunctioning of the lateral PFC to its entire volume and supporting again the assumption of hypoactivity of key regions during eating control in obese individuals.

On the other hand, Levy and Wagner's meta-analysis (90) revealed that the middle part of the right ventrolateral PFC (BA45) is involved in decision uncertainty. This function was found to be present only in the right ventrolateral PFC. Interestingly, Hsu et al. (49) observed that during general inhibition (i.e., non-food specific), the right BA45 was significantly more activated among obese than lean participants. Corroborating the results from Scharmüller et al. (52) in BA8, we propose that this finding may connote a decreased ability to firmly make the choice to inhibit from acting in obese individuals. However, the site of increased activation within the right BA45 was on the border of the insular region. Considering the various preprocessing steps taken to analyze fMRI data (i.e., realignment, normalization, and smoothing), the accuracy of the obtained sites of activation and related coordinates through the MRI methods bear some inaccuracies. Our assumption of greater levels of uncertainty linked to obesity should then be taken precociously, as the results from Hsu et al. (49) may also reflect insular hyperactivity.

#### Premotor Cortex

Situated in the superior part of the frontal cortex, Brodmann area (BA) 6 is located on the posterior part of the premotor cortex and corresponds to the supplementary motor area, also described as secondary motor cortex. Several behavioral tasks, such as the go/no-go and stop-signal task, emphasize its importance for response inhibition (85, 91, 92). In individuals with substance addiction, decreased activation of the supplementary motor area has been observed during those two inhibition tasks (93, 94). Considering the neural similarities as well as the comparable exacerbated levels of impulsivity in individuals with substance and food addiction (19, 95), results from Batterink et al. (43) and Hendrick et al. (48) are not surprising. These authors reported that, when compared with their lean counterparts, obese individuals exhibited hypoactivity of the supplementary motor area during both general and food-specific inhibition processes.

### Limbic Regions

The limbic system is composed of a set of interconnected subcortical but also cortical structures. Those numerous connections form complex circuits (96), known to be highly involved in the regulation of emotion-related behavior (97).

In the present review, we report the major contributions of the limbic system to eating behavior control, but impaired among the obese population, either showing hypo- or hyperactivity in different limbic regions according to their functional role in inhibitory control (Figure 3).

#### Insula

As part of the gustatory cortex (98), the insula is known to play a role in smell and taste processing, as well as in fat detection (63). The insula is also a crucial region for homeostatic regulation, with its external input and expected reward integration function (99). It was more recently suggested that through the translation of objective interoceptive signals into subjective experiences such as craving, which will potentiate impulsivity, the insula may play a role in the onset and maintenance of addiction (24). For instance, experimentally silenced insula in rats and lesioned insula in humans were found to be, respectively, associated with amphetamine (100) and smoking craving disruption (101).

Consistent with the results suggesting increased insular activations during inhibition to be characteristic of inhibitory control difficulties (102) and to correlate with the tendency to eat in response to food stimuli regardless of the state of hunger in obese adolescents (103), the articles we reviewed show a hyperactivity of the insula (BA13) in obese in comparison with lean participants when inhibiting (32, 43) (see further section for the inverted U-shaped activation of the insula in relation to BMI). Only Hendrick et al. (48) found an opposite pattern of activity. However, the site of activation was on the boarder of the OFC. For the same methodological reason we mentioned before (i.e., spatial resolution accuracy), the hypoactivity found by Hendrick et al. (48) in the part of the insula bordering the OFC could potentially be attributed to the lateral OFC rather than to the insular cortex.

#### Cingulate Cortex

##### Posterior Cingulate Cortex

The posterior part of the cingulate cortex is involved in processing emotionally relevant stimuli and memory-related functions (104, 105). Interestingly, its level of activation during high-calorie food anticipation was found to be associated with BMI (106). Results from Tuulari et al. (53), showing a hyperactivity of this region in obese participants in comparison with their lean counterparts, may therefore be the neural signature of excessive episodic memory-related hyperactivity during eating anticipation based on constrained downregulation and, hence, inhibition capabilities in obese individuals.

##### Anterior Cingulate Cortex

Besides forming an integral part of the limbic system, the anterior cingulate cortex is often considered as belonging to the frontal cortex (107) and associated inhibitory control networks (108, 109). Playing a major role in palatable food salience attribution and subsequent decision-making (63), the anterior cingulate cortex was demonstrated to be involved in the regulation of food craving and to be related to BMI. Specifically, He et al. (46) and Giuliani et al. (80) reported a negative correlation between anterior cingulate cortex activations and BMI during a food-related inhibition task. In line with those results, Tuulari et al. (53) showed that obese participants exhibited significantly lower activation in this region, therefore suggesting an impaired palatable food salience attribution with consequences on food choices in case of obesity.

#### Thalamus

The thalamus has been suggested to be involved in substance addiction due to its function in expectation processing (110). Expectation of the rewarding effects of drug consumption is thought to be responsible for the reinforcing dynamic of drug abuse (111). The role of the thalamus in the context of food addiction is nonetheless less elucidated. A part of the thalamus, namely the paraventricular thalamus, has been postulated to be a gateway to feeding and appetitive motivation. Specifically, it was suggested that its strategic position between brain regions responsible for homeostatic perception (i.e., hindbrain and hypothalamus), motivation, and reward processes (i.e., amygdala, ventral striatum, and cortex), allows the paraventricular thalamus to control eating behavior *via* bottom-up and top-down control (112). The importance of this part of the thalamus for controlling food intake was evidenced by animal research, revealing that lesions (113), pharmacological activation of its GABA_A_ receptors (114), or its chemogenetic inhibition (115) causes increased food intake. On the contrary, activation of the paraventricular neurons was found to reduce food intake (115). Assuming that the results observed in mice would apply to human, we propose that thalamic hypoactivity in obese individuals (49) may reflect a dysfunctional inhibition of eating.

#### Caudate Nucleus

Together with the anterior part of the putamen, the head of the caudate nucleus represents the dorsal striatum, a region primarily responsible for reward processing (116). The mere exposure to food items is enough to produce dopamine release in this brain region (117). In the addiction literature, the caudate nucleus function is also linked to impulsivity. For instance, decreased caudate nucleus activations during the reception of a pleasant taste were associated with impulsivity and obesity (118, 119). Obesity-related lower dopamine signaling during the ingestion of food refers to the “reward-deficiency” theory, justifying the need for compensatory overeating to trigger satisfying reward responses (67). To explain the hypoactivity of the caudate nucleus during the attempt to inhibit from craving in their obese vs. lean participants, Tuulari et al. (53) referred to the reward deficiency theory. However, one may also argue that a hypoactive reward system would rather lead to improved inhibitory control. In this context, it is interesting to refer to the implication of the caudate nucleus in motor inhibition (120–122). In the study by Tuulari et al. (53), the dorsal part of the caudate nucleus was found to be hypoactive. While the ventral caudate, mainly interconnected with the limbic system, is involved in the processing of affects (123), the dorsal caudate is connected to the motor, cingular, and dorsolateral prefrontal cortices (124–126). Furthermore, it is thought to play a role not only in motor but also executive functions (127, 128). Therefore, an alternative interpretation of the hypoactivation of the dorsal caudate nucleus during attempting to inhibit the urge to eat in obese individuals could originate from impaired motor and/or cognitive inhibitory functions.

#### Parahippocampal Gyrus

As part of the reward system, the parahippocampal gyrus is involved in hedonic feeding and incentive motivation processes (129). Its activity was shown to be responsive to the perception of food items (130) and to correlate positively with obesity (9) and weight gain (131). The role of the parahippocampal gyrus during reward processing seems to depend on its function in emotional memory (129, 132). In Chen et al. (133), trait-based food craving was associated with spontaneous neuronal activity in the parahippocampal gyrus. This suggests reinforced food-related hedonic memories in obese individuals. However, the hypersensitivity of the food reward system is rather counterintuitive to explain the results from Hsu et al. (49), reporting a hypoactivity of the right parahippocampal gyrus (BA36) during inhibition among obese in comparison with lean individuals.

Besides its role in reward and memory functions, the parahippocampal gyrus also seems to underpin inhibition. For instance, using the go/no-go task in healthy adults, Nakata et al. (134) revealed the implication of this brain region in inhibition processes. Its activity was further shown to positively correlate with inhibition success (135). During no-go trials, normally developing children activated a neural network also comprising the parahippocampal gyrus, whereas children with attention deficit/hyperactivity disorder, which is associated with impaired inhibition capacities (136), failed to activate this region during inhibition attempts (137). In the literature specific to addiction, Sheinkopf et al. (138) revealed that activations of the parahippocampal gyrus during inhibition were significantly attenuated in children with prenatal exposure to cocaine. Despite the lack of information specifically concerning the role of this region in inhibition processes in individuals with food addiction, we propose that the hypoactivity observed by Hsu et al. (49) during no-go trials is likely to reflect an impairment of the inhibitory function hosted by the parahippocampal gyrus in the obese population.

### Additional Regions

#### Visual Cortex

**Figure 4:**
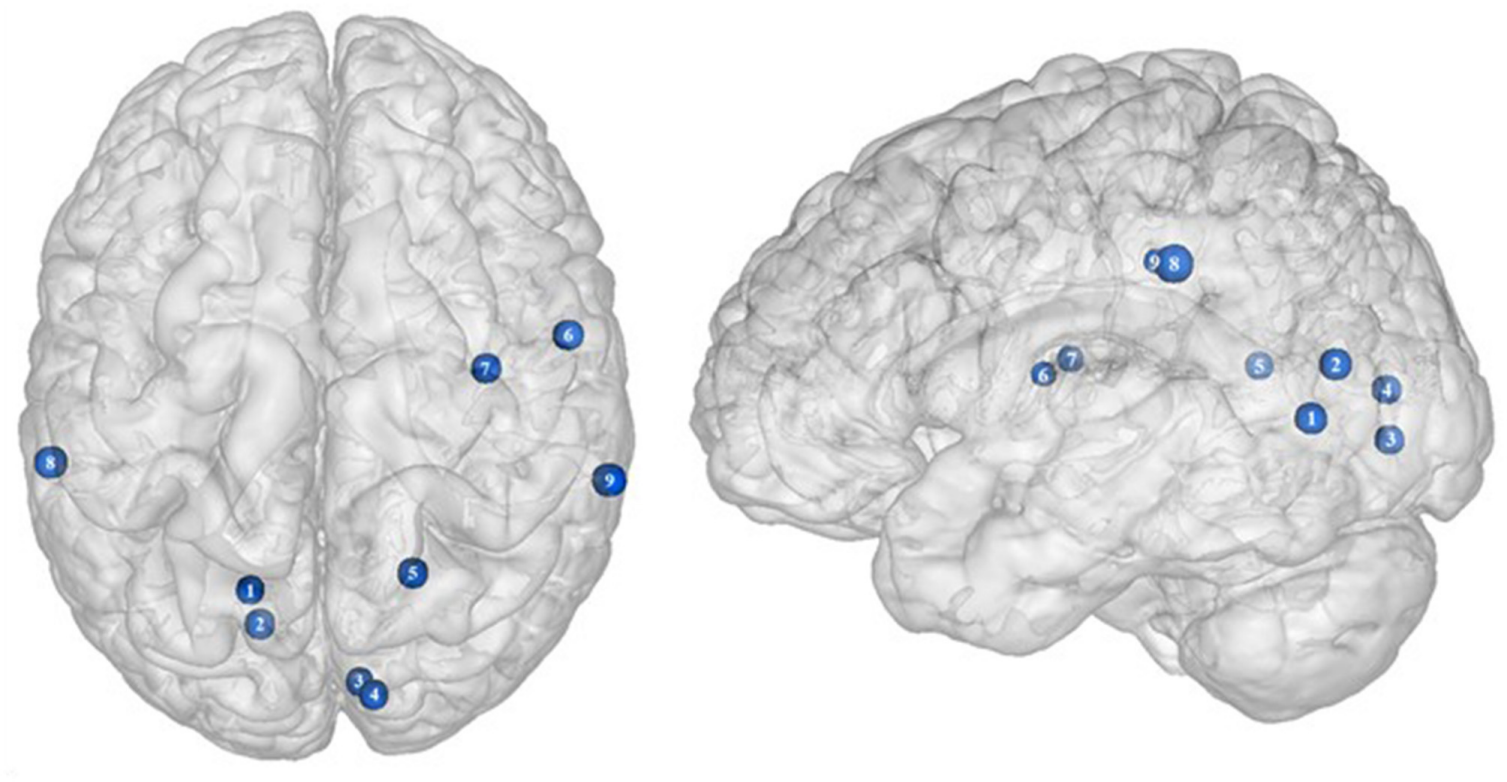
Functional activity in other regions during inhibition. Blue color indicates hypoactivity in obese in comparison with lean individuals during inhibition (see Table 4 for precise coordinates). 1–5 = visual cortex (48), 6–7 = rolandic operculum (49), 8–9 = inferior parietal cortex (48).

Located in the posterior part of the occipital cortex, the cuneus belongs to the visual cortex. Besides its essential role in the processing of visual information (139), the cuneus has been shown to be involved in addiction. Both structural and functional impairments of the cuneus have been suggested to be associated with addiction disorders. For instance, decreased cuneus volume was found to negatively correlate with years of drug use in cocaine addicts (140) and to predict relapse in alcoholics (141). In the same vein, using a color-word drug Stroop task, Goldstein et al. (142) found hypoactivation of the cuneus during inhibition in cocaine addicts. Although the structural and functional properties of the cuneus in individuals with food addiction remain to be elucidated, we propose that the hypoactivity reported by Hendrick et al. (48) in obese in comparison with lean individuals during inhibition (Figure 4) may stem from altered cuneus volume and/or function.

#### Rolandic Operculum

Studies investigating the neural responses to both food anticipation and delivery revealed a hyper-responsivity of the Rolandic operculum in obese adults (143) and adolescents (144), hence suggesting this brain region to be part of the food reward system. Decreased activations of the Rolandic operculum should therefore concur with decreased reward responses, hence facilitating inhibition processes. In that sense, its hypoactivity reported by Hsu et al. (49) in obese individuals during inhibition may seem counterintuitive. This result rather suggests that the Rolandic operculum may actively play a role not only in reward but also in inhibition processes related to food behavior. In drug addicts, studies reported decreased gray matter volume in this brain region (145, 146). Likewise, using single-photon emission computed tomography, Willeumier et al. (147) scanned a cohort of suicide, also known to present excessive levels of impulsivity (148). The authors revealed decreased cerebral blood flow in the region of the Rolandic operculum. Considering those results, we assume that the hypoactivity of the Rolandic operculum during inhibition among obese individuals (49) (Figure 4) may stem from hypoperfusion and/or atrophy, resulting from high impulsivity levels and, in turn, leading to inadequate response inhibition.

#### Inferior Parietal Cortex

The inferior parietal cortex has been shown to be consistently involved in response inhibition processes, measured either with go/no-go (149), stop signal reaction time (150), or other adapted tasks (151, 152). Interestingly, in populations showing high levels of impulsivity, such as individuals with attention deficit/hyperactivity disorder and alcoholics, voxel-based morphometry analyses showed decreased gray matter volume of the inferior parietal cortex (153, 154). The deleterious effects of impulsivity were also found to not only impact inferior parietal cortex structure but also its functionality. Notably, Schilling et al. (155) reported decreased gray matter volumes and Horn et al. (102) showed decreased neural activity during no-go trials. In the specific context of obesity, Stoeckel et al. (156) revealed that the inferior parietal cortex was less activated during difficult than easy delay-discounting trials. To that extent, we propose that the hypoactivity of the inferior parietal cortex (BA40) reported by Hendrick et al. (48) in obese individuals during stop signals (Figure 4) reflects the impairment of inhibitory capacities and, hence, the amplification of impulsivity, probably as a consequence of structural and/or functional alterations.

#### Inverted U-Shaped Reward Activations

**Figure 5:**
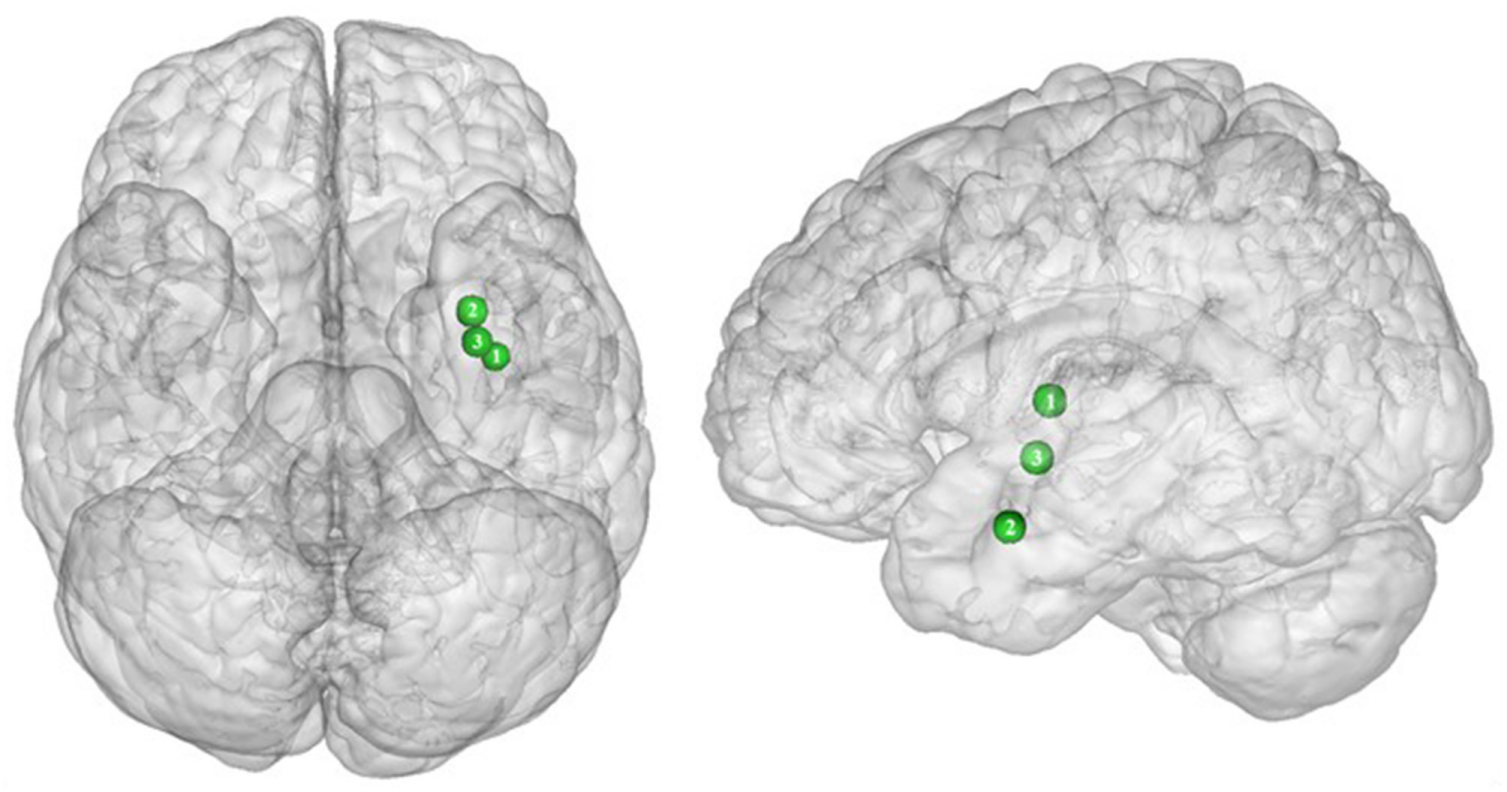
Inverted U-shaped pattern of functional activity during inhibition. Green color indicates an increased neural activity in class-I obese (BMI = 30) in comparison with lean participants, and an attenuated activity in obesity classes II and III (BMI = 35–40) reaching similar patterns of activation as in lean participants (see Table 5 for precise coordinates). 1 = insula (32), 2 = claustrum (32), 3 = Putamen (32).

In their study, Dietrich et al. (32) found an inverted U-shaped pattern of activation in the insula, claustrum, and putamen during inhibition (Figure 5), with the highest activation values observed among class I obese participants (BMI = 30). With its role merging the detection of food characteristics and interoception, the insula plays a major role in reward processing. Increased insular activations in class I obese participants support the assumption of a hypersensitivity of the reward system in addicts (13, 95, 144). A similar pattern of activation was obtained from the putamen, which together with the caput caudatum constitutes the ventral striatum, a key region of the brain's reward system (157). The putamen entails both the delivery and the detection of reward (158) and is part of the corticolimbic pathway controlling food reward processes (67). Moreover, supporting habit formation (159), the putamen is involved in the mediation of habitual eating behavior (67). The creation of a habit, originating from previous goal-directed behavior, is accelerated through dopamine release (160). Obesity, and thus habitual overeating, was found to be positively associated with dopamine D2-like receptors in the putamen. This suggests that habitual excessive eating behavior observed in obese individuals may partly stem from the putamen's sensitivity to enhanced dopamine release. Another explanation for this increased activation of the putamen in class I obese individuals during inhibition is that highly caloric foods act as strong reward reinforcers (19) and may thus hamper automatic eating behavior control, hence leading to overeating (161). This loss of control resulting from exposure to salient stimulus refers to an attentional bias. In individuals with substance addiction, attentional bias was found to positively correlate with craving and putamen activations (162).

Between the insula and the putamen lies the claustrum, a very small brain region [i.e., roughly 0.25% of the cerebral cortex (163)], but nonetheless highly connected (164). It was found to be also hyperactive among class I obese. Due to its bilateral connections with all areas of the cortex, the claustrum has been identified as a key node for multisensory integration and then responsible for the encoding of stimuli salience (165). To reduce the affluence of cortical information not selected for attention, the claustrum operates *via* a selective activation of inhibitory neurons in layer IV (166). The hyperactivity of this region among obese individuals during inhibition could be the sign of an exacerbated inhibitory activity aiming at focusing attention through compensating the excessive intensity of perception-related cortical processing. This hypothesis would reflect an excessive sensorial input and/or difficulty in deciding for the salience qualities of a given food-related input in class I obese individuals.

Altogether, those results support the notion of a hypersensitive reward system in obese individuals, but only partially. While activation in these areas linearly increases up to a BMI of 30, a BMI from 30 to 40 is associated with the opposite dynamic, suggesting a decreased hypersensitivity of the reward system in class II obese individuals. Parallel to reward hypersensitivity, the reward hyposensitivity hypothesis can also be found in the addiction literature [i.e., reward deficiency theory (95, 144)]. While the former applies during eating anticipation, the later occurs during actual reception. According to the reward deficiency theory, obese individuals must consume more food to reach satisfying reward activations and subsequent pleasure perception (67). Neurobiological evidence for this assumption relies on decreased dopamine D2 receptor availability in obese individuals' brain (167). Results from Dietrich et al. (32) suggest that this alteration of the dopaminergic system may be BMI dependent and within the different obese classes. The inverted U-shaped pattern of activation these authors found in the insula, putamen, and claustrum could signify that the dynamics of the impaired dopaminergic system may be reversed with increasing obesity severity and that the reward deficiency theory also applies to food anticipation in severely obese individuals.

### Functional Connectivity

**Figure 6:**
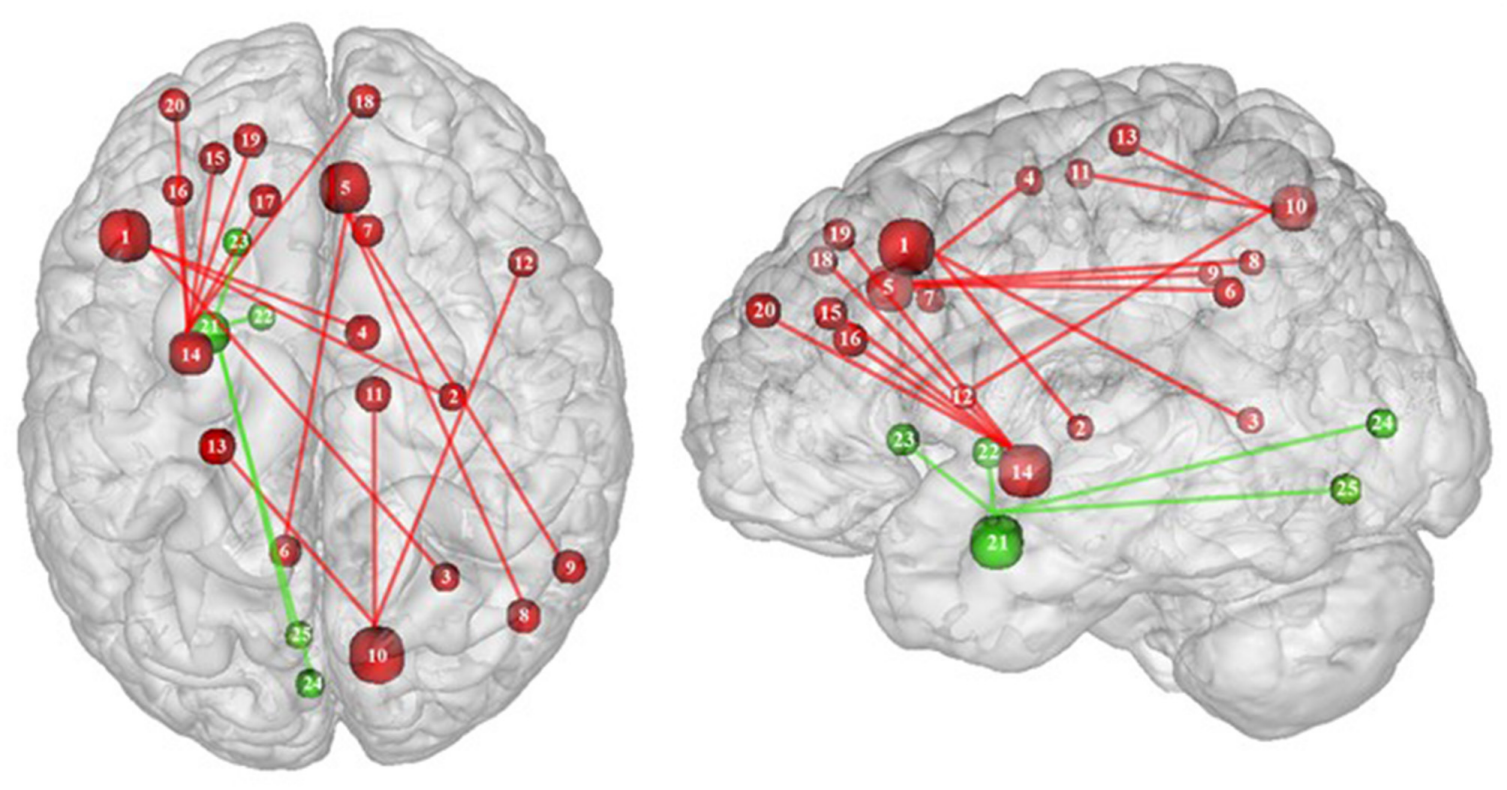
Functional connectivity during inhibition. Red and green connections indicate hyperconnectivity and U-shaped connectivity, respectively, in obese in comparison with lean participants during inhibition (see Table 7 for precise coordinates). Regions n°1, 5, 10, 14, and 21 are seed regions. 1 = dorsolateral prefrontal cortex, 2 = putamen, 3 = cingulate cortex, 4 = supplementary motor area, 5 = pre-supplementary motor area, 6 = precuneus, 7 = cingulate cortex, 8–9 = parietal cortex, 10 = precuneus, 11 = supplementary motor area, 12 = ventrolateral prefrontal cortex, 13 = sensory cortex (53), 14 = putamen, 15–19 = dorsolateral prefrontal cortex, 20 = dorsomedial prefrontal cortex, 21 = amygdala, 22 = pallidum, 23 = putamen, 24–25 = visual cortex (32).

Functional connectivity studies showed a stronger connectivity of frontal regions (dorsolateral PFC and pre-supplementary motor area) with the putamen, cingulate cortex, supplementary motor area, precuneus, and inferior parietal cortex (53) (Figure 6), suggesting a shift in neural demand away from executive to hedonic functions during inhibition of food intake. This strengthened functional connectivity between inhibition and reward-related nodes was furthermore found to be a two-way relationship, notably between the inferior parietal cortex (precuneus), the basal ganglia (putamen), and frontal regions (dorsolateral PFC, dorsomedial PFC, ventrolateral PFC, and supplementary motor area) (32, 53). As the putamen is involved in the attribution of food reward value and in the mediation of habitual eating behavior (see previous section), and the precuneus plays a role in a large number of affective and cognitive functions (168), the strengthened functional connectivity between those reward regions with the frontal part of the brain may also reflect increased neural demand for the successful control of food intake in the obese population. The hyperactive interplay between frontal and limbic regions during inhibition in obese individuals may be the adaptive signature of increased neural activity aiming at compensating the pathologically induced decreased functioning of those regions.

Conversely, a U-shaped pattern of functional connectivity was reported between the amygdala, involved in motivational salience encoding (169), and regions involved in reward perception and signaling (32) (Figure 6). Whereas, the functional activity in lean and severely obese individuals was found to be similar, decreased values were reported in class I obese individuals between the amygdala, visual cortex, and basal ganglia (pallidum and putamen). Dietrich et al. (32) assumed that BMI may affect craving regulation through a U-shaped modulation of the interplay between salience encoding and pleasantness computation. This would mean that whereas mildly obese individuals show a decreased functional connectivity of the regions involved in craving regulation, morbidly obese individuals' neural activity is similar to the pattern obtained from normal weighted controls, despite an exacerbated behavioral loss of control regarding food craving regulation (probably even greater than among mildly obese individuals). This assumption seems counterintuitive to us. We therefore assume that the U-shaped pattern of functional connectivity found in this brain network may rely on other explanations, however still unknown and deserving further examination.

### Binge Eating Disorder

**Figure 7:**
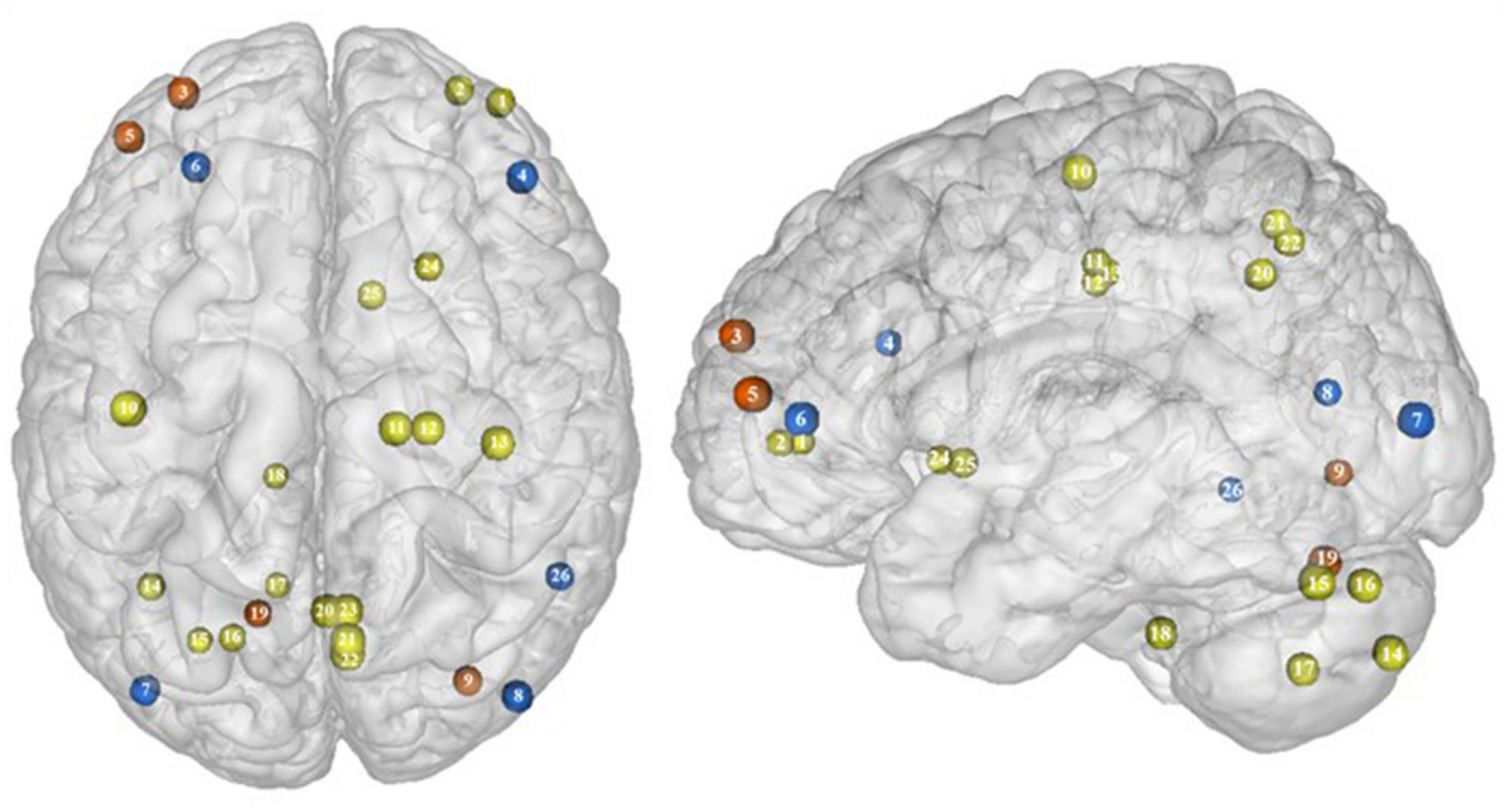
Functional activity in individuals with binge eating disorder during inhibition. Blue color indicates hypoactivity in obese individuals with binge eating disorder in comparison with obese individuals without binge eating disorder during inhibition. Yellow and orange colors indicate hypo- and hyperactivity, respectively, in lean with binge eating disorder in comparison with lean without binge eating disorder (see Table 6 for precise coordinates). 1–2 = ventromedial prefrontal cortex (51), 3 = dorsomedial prefrontal prefrontal cortex (51), 4 = dorsolateral prefrontal cortex (47), 5 = dorsolateral prefrontal cortex (51), 6 = ventrolateral prefrontal cortex (44), 7–8 = visual cortex (44), 9 = visual cortex (51), 10 = primary motor cortex (51), 11–12 = premotor cortex (51), 13 = primary sensory motor cortex (51), 14–19 = cerebellum (51), 20–23 = precuneus (51), 24–25 = putamen (51), 26 = temporal gyrus (44).

Part of the obese population is confronted with an aggravated form of excessive eating behavior, consisting in consuming a large amount of food within a short period of time, paired with a sensation of loss of control. This pathological eating behavior, named BED, is referenced as a stand-alone illness in the DSM-5 (170). As binge period may be followed by purge (e.g., vomiting), not all individuals suffering from BED are obese. This is however true in 40% of the cases (171). Importantly, in comparison with obese individuals without BED, those presenting the pathology were found to have higher psychiatric comorbidities and diabetes rates, as well as more physical symptom and health dissatisfaction (172–175). Considering those alarming observations, a deeper understanding of the disinhibition processes underlying BED, and specifically among the obese population, is critical. As behavioral impulsivity was found to be even more pronounced among obese with BED (176, 177), it can be expected that the neural networks responsible for inhibition, already found to be downregulated in the obese population (see previous sections), are presenting further weakening in the presence of BED. Results from Balodis et al. (44) and Hege et al. (47) support this assumption. Using food-specific Stroop color-word interference and go/no-go tasks, these authors emphasize the exacerbated decrease of frontal regions' activity. Specifically, obese individuals with BED showed less neural activation within the ventrolateral (BA46) and dorsolateral part (BA9) of the prefrontal cortex. The same impairment was also observed in the visual cortex, more precisely in the cuneus (BA19). Besides this exacerbation of the frontal and visual regions' hypoactivity, the study from Balodis et al. (44) further revealed hypofunctioning of the middle (BA21) and inferior parts (BA37) of the temporal lobe. Belonging to the cognitive system (178, 179), the temporal lobe is also involved in inhibition processes (180, 181). Higher impulsivity levels have been found to be associated with reduced gray matter volume (182) and regional cerebral blood flow (183) in the temporal lobe. Moreover, animal studies showed that structural alterations of the temporal cortex led to hyperphagia and obesity (184). This relation between temporal cortex integrity and eating behavior was also observed among humans. Gray matter volume was notably found to be negatively correlated with BMI (185, 186) and, accordingly, systematically reduced in obese individuals (187–189). A 24-year longitudinal study showed that individuals who were starting to increase their BMI during their middle age present decreased gray matter volume in the temporal lobe at a later age (190). This suggests that this brain alteration is a consequence from obesity, probably because of related insulin resistance (15). However, women with an atrophy of the temporal lobe were found to have a higher BMI (190), here suggesting the reversed cause–effect relationship between obesity and the temporal lobe. In essence, results from Balodis et al. (44) and Hege et al. (47) support the assumption of similar, although exacerbated, as well as further inhibition network impairments in obese with binge eating disorder (Figure 7).

Binge eating episodes can also occur in normal-weight individuals (191). They are thought to occur more frequently and compulsively in the course of time (192), hence increasing the risk to develop obesity. To provide a better understanding of the tendency to binge eating, Oliva et al. (51) investigated neural activity of normal-weight adults while performing the go/no-go and stop signal inhibition tasks. Inconsistent with the results from Hege et al. (47) who used the classical version of the go/no-go task in obese with BED, Oliva et al. (51) reported increased activity of the dlPFC during the food-specific version of the stop signal task. Those results are however consistent with the findings from Scharmüller et al. (52) who also used a food-specific inhibition task. This suggests that the opposite patterns of activity observed in the dlPFC in lean individuals with BED in comparison with obese individuals with BED may not only steam from the presence of obesity but also from the nature of the inhibition task. This may also explain the increased activity observed in the visual cortex in lean individuals with BED during inhibition, whereas those with BED and obesity were shown to present hypoactivity (44). Besides those incongruent results with obese BED, lean BED further showed hypoactivity of the primary sensorimotor cortex, precuneus, putamen, and cerebellum, all being involved in inhibition processes (59, 193–195). To what extent an impairment of those regions during inhibition may represent early warning signs of obesity development deserves further longitudinal investigation.

## Limitations

This review article entails studies that used general as well as food-specific paradigms to investigate inhibition processes, despite coming from different methodological paradigms (i.e., experimental tasks). Thus, this precludes obtaining information on the general or specific aspect of inhibition impairment in individuals with obesity and BED. Moreover, only 12 studies could be included, hence not allowing detailed and firm conclusions to be drawn. That is why we aimed at additionally adding EEG studies. However, all studies we found, although they showed impaired inhibition response, did not report on precise brain regions. Thus, we did not include EEG studies in this review.

## Conclusion

In this review, we gathered further evidence of shared neural impairment underlying drug and food addiction, mainly targeting the frontal and limbic regions. Specifically, obesity is linked to alterations of the dopaminergic system during response inhibition, translating into hypoactivity of the frontal regions and either hypo- or hyperactivity of the limbic regions according to their role in behavior control. We further conclude that the presence of BED may lead to similar although greater impairment of the inhibition system. We however suggest that a deeper and more grounded understanding of the inhibition system impairment related to obesity should constitute the subject matter of future studies. Investigating whether alterations of the dopaminergic function and its related inhibition processes in obese individuals are rather general or specific to eating behavior, and also whether it varies within different classes of obesity, could provide valuable insights for the comprehension of inhibition impairment in the obese population.

In summary, inhibition processes are found to rely on a complex neural network involving the prefrontal but also the frontal and subcortical regions. Whether stimulation of the identified key areas, such as the striatum, orbitofrontal, medial prefrontal, cingulate, or insular cortex, may lead to substantial improved eating control deserves future non-invasive (transcranial direct current stimulation, repetitive transcranial magnet stimulation) as well as invasive brain stimulation studies (deep brain stimulation).

## Data Availability Statement

The original contributions presented in the study are included in the article/supplementary material, further inquiries can be directed to the corresponding author/s.

## Author Contributions

ES wrote the manuscript. BP revised the manuscript. Both authors contributed to the article and approved the submitted version.

## Conflict of Interest

The authors declare that the research was conducted in the absence of any commercial or financial relationships that could be construed as a potential conflict of interest.

## References

[1] Volkow ND O'Brien CP. Issues for DSM-V: should obesity be included as a brain disorder? Am Psychiatric Assoc. (2007) 164:708–10. 10.1176/ajp.2007.164.5.708 17475727

[2] Levine AS Kotz CM Gosnell BA. Sugars: hedonic aspects, neuroregulation, and energy balance. Am J Clin Nutr. (2003) 78:834S–42. 10.1093/ajcn/78.4.834S 14522747

[3] Lenoir M Serre F Cantin L Ahmed SH. Intense sweetness surpasses cocaine reward. PLoS ONE. (2007) 2:e698. 10.1371/journal.pone.0000698 17668074 PMC1931610

[4] Weltens N Zhao D Van Oudenhove L. Where is the comfort in comfort foods? Mechanisms linking fat signaling, reward, and emotion. Neurogastroenterol Motil. (2014) 26:303–15. 10.1111/nmo.12309 24548257

[5] Volkow ND Wise RA. How can drug addiction help us understand obesity? Nat Neurosci. (2005) 8:555–60. 10.1038/nn1452 15856062

[6] DiFeliceantonio AG Small DM. Dopamine and diet-induced obesity. Nat Neurosci. (2019) 22:1–2. 10.1038/s41593-018-0304-0 30559474

[7] Volkow ND Wang G-J Fowler JS Tomasi D Telang F Baler R. Addiction: decreased reward sensitivity and increased expectation sensitivity conspire to overwhelm the brain's control circuit. BioEssays News Rev Mol Cell Dev Biol. (2010) 32:748–55. 10.1002/bies.201000042 20730946 PMC2948245

[8] Guzzardi MA Iozzo P. Brain functional imaging in obese and diabetic patients. Acta Diabetol. (2019) 56:135–44. 10.1007/s00592-018-1185-0 29959509

[9] Brooks SJ Cedernaes J Schiöth HB. Increased prefrontal and parahippocampal activation with reduced dorsolateral prefrontal and insular cortex activation to food images in obesity: a meta-analysis of fMRI studies. PLoS ONE. (2013) 8:e60393. 10.1371/journal.pone.0060393 23593210 PMC3622693

[10] Corsica JA Pelchat ML. Food addiction: true or false? Curr Opin Gastroenterol. (2010) 26:165–9. 10.1097/MOG.0b013e328336528d 20042860

[11] Gearhardt AN Yokum S Orr PT Stice E Corbin WR Brownell KD. Neural correlates of food addiction. Arch Gen Psychiatry. (2011) 68:808–16. 10.1001/archgenpsychiatry.2011.32 21464344 PMC3980851

[12] Stice E Figlewicz DP Gosnell BA Levine AS Pratt WE. The contribution of brain reward circuits to the obesity epidemic. Neurosci Biobehav Rev. (2013) 37:2047–58. 10.1016/j.neubiorev.2012.12.001 23237885 PMC3604128

[13] Stoeckel LE Weller RE Cook EW Twieg DB Knowlton RC Cox JE. Widespread reward-system activation in obese women in response to pictures of high-calorie foods. NeuroImage. (2008) 41:636–47. 10.1016/j.neuroimage.2008.02.031 18413289

[14] Mela DJ. Eating for pleasure or just wanting to eat? Reconsidering sensory hedonic responses as a driver of obesity. Appetite. (2006) 47:10–7. 10.1016/j.appet.2006.02.006 16647788

[15] Maayan L Hoogendoorn C Sweat V Convit A. Disinhibited eating in obese adolescents is associated with orbitofrontal volume reductions and executive dysfunction. Obesity. (2011) 19:1382–7. 10.1038/oby.2011.15 21350433 PMC3124611

[16] Lavagnino L Arnone D Cao B Soares JC Selvaraj S. Inhibitory control in obesity and binge eating disorder: a systematic review and meta-analysis of neurocognitive and neuroimaging studies. Neurosci Biobehav Rev. (2016) 68:714–26. 10.1016/j.neubiorev.2016.06.041 27381956

[17] Dillon DG Pizzagalli DA. Inhibition of action, thought, and emotion: a selective neurobiological review. Appl Prev Psychol. (2007) 12:99–114. 10.1016/j.appsy.2007.09.004 19050749 PMC2396584

[18] Ridderinkhof KR Van Den Wildenberg WP Segalowitz SJ Carter CS. Neurocognitive mechanisms of cognitive control: the role of prefrontal cortex in action selection, response inhibition, performance monitoring, and reward-based learning. Brain Cogn. (2004) 56:129–40. 10.1016/j.bandc.2004.09.016 15518930

[19] Volkow ND Wang G-J Fowler JS Telang F. Overlapping neuronal circuits in addiction and obesity: evidence of systems pathology. Philos Trans R Soc B Biol Sci. (2008) 363:3191–200. 10.1098/rstb.2008.0107 18640912 PMC2607335

[20] Volkow ND Fowler JS. Addiction, a disease of compulsion and drive: involvement of the orbitofrontal cortex. Cereb Cortex. (2000) 10:318–25. 10.1093/cercor/10.3.318 10731226

[21] Volkow ND Wang G-J Begleiter H Porjesz B Fowler JS Telang F. High levels of dopamine D2 receptors in unaffected members of alcoholic families: possible protective factors. Arch Gen Psychiatry. (2006) 63:999–1008. 10.1001/archpsyc.63.9.999 16953002

[22] Fineberg NA Potenza MN Chamberlain SR Berlin HA Menzies L Bechara A. Probing compulsive and impulsive behaviors, from animal models to endophenotypes: a narrative review. Neuropsychopharmacol Off Publ Am Coll Neuropsychopharmacol. (2010) 35:591–604. 10.1038/npp.2009.185 19940844 PMC3055606

[23] Jentsch JD Taylor JR. Impulsivity resulting from frontostriatal dysfunction in drug abuse: implications for the control of behavior by reward-related stimuli. Psychopharmacology. (1999) 146:373–90. 10.1007/PL00005483 10550488

[24] Chen R Li DP Turel O Sørensen TA Bechara A Li Y. Decision making deficits in relation to food cues influence obesity: a triadic neural model of problematic eating. Front Psychiatry. (2018) 9:264. 10.3389/fpsyt.2018.00264 29962976 PMC6010920

[25] Murphy CM Stojek MK MacKillop J. Interrelationships among impulsive personality traits, food addiction, and body mass index. Appetite. (2014) 73:45–50. 10.1016/j.appet.2013.10.008 24511618 PMC4859335

[26] Calvo D Galioto R Gunstad J Spitznagel MB. Uncontrolled eating is associated with reduced executive functioning. Clin Obes. (2014) 4:172–9. 10.1111/cob.12058 25826773

[27] Houben K Nederkoorn C Jansen A. Eating on impulse: the relation between overweight and food-specific inhibitory control. Obesity. (2014) 22:E6–8. 10.1002/oby.20670 24910860

[28] Logan GD Schachar RJ Tannock R. Impulsivity and inhibitory control. Psychol Sci. (1997) 8:60–4. 10.1111/j.1467-9280.1997.tb00545.x

[29] Guerrieri R Nederkoorn C Stankiewicz K Alberts H Geschwind N Martijn C. The influence of trait and induced state impulsivity on food intake in normal-weight healthy women. Appetite. (2007) 49:66–73. 10.1016/j.appet.2006.11.008 17261343

[30] Kamijo K Pontifex MB Khan NA Raine LB Scudder MR Drollette ES. The association of childhood obesity to neuroelectric indices of inhibition. Psychophysiology. (2012) 49:1361–71. 10.1111/j.1469-8986.2012.01459.x 22913478

[31] Nederkoorn C Smulders FTY Havermans RC Roefs A Jansen A. Impulsivity in obese women. Appetite. (2006) 47:253–6. 10.1016/j.appet.2006.05.008 16782231

[32] Dietrich A Hollmann M Mathar D Villringer A Horstmann A. Brain regulation of food craving: relationships with weight status and eating behavior. Int J Obes. (2016) 40:982–9. 10.1038/ijo.2016.28 26883294

[33] Mole TB Irvine MA Worbe Y Collins P Mitchell SP Bolton S. Impulsivity in disorders of food and drug misuse. Psychol Med. (2015) 45:771–82. 10.1017/S0033291714001834 25118940 PMC4998952

[34] Weller RE Cook EW Avsar KB Cox JE. Obese women show greater delay discounting than healthy-weight women. Appetite. (2008) 51:563–9. 10.1016/j.appet.2008.04.010 18513828

[35] Schag K Schönleber J Teufel M Zipfel S Giel KE. Food-related impulsivity in obesity and binge eating disorder–a systematic review. Obes Rev. (2013) 14:477–95. 10.1111/obr.12017 23331770

[36] Giel K Teufel M Junne F Zipfel S Schag K. Food-related impulsivity in obesity and binge eating disorder—a systematic update of the evidence. Nutrients. (2017) 9:1170. 10.3390/nu9111170 29077027 PMC5707642

[37] Pull CB. Binge eating disorder. Curr Opin Psychiatry. (2004) 17:43–8. 10.1097/00001504-200401000-00008

[38] Pleger B. Invasive and non-invasive stimulation of the obese human brain. Front Neurosci. (2018) 12:884. 10.3389/fnins.2018.00884 30555295 PMC6281888

[39] Goldman RL Borckardt JJ Frohman HA O'Neil PM Madan A Campbell LK. Prefrontal cortex transcranial direct current stimulation (tDCS) temporarily reduces food cravings and increases the self-reported ability to resist food in adults with frequent food craving. Appetite. (2011) 56:741–6. 10.1016/j.appet.2011.02.013 21352881

[40] Kekic M McClelland J Campbell I Nestler S Rubia K David AS. The effects of prefrontal cortex transcranial direct current stimulation (tDCS) on food craving and temporal discounting in women with frequent food cravings. Appetite. (2014) 78:55–62. 10.1016/j.appet.2014.03.010 24656950

[41] Harat M Rudaś M Zieliński P Birska J Sokal P. Nucleus accumbens stimulation in pathological obesity. Neurol Neurochir Pol. (2016) 50:207–10. 10.1016/j.pjnns.2016.01.014 27154450

[42] Whiting DM Tomycz ND Bailes J de Jonge L Lecoultre V Wilent B. Lateral hypothalamic area deep brain stimulation for refractory obesity: a pilot study with preliminary data on safety, body weight, and energy metabolism. J Neurosurg. (2013) 119:56–63. 10.3171/2013.2.JNS12903 23560573 PMC5666570

[43] Batterink L Yokum S Stice E. Body mass correlates inversely with inhibitory control in response to food among adolescent girls: an fMRI study. Neuroimage. (2010) 52:1696–703. 10.1016/j.neuroimage.2010.05.059 20510377 PMC2910204

[44] Balodis IM Molina ND Kober H Worhunsky PD White MA Sinha R. Divergent neural substrates of inhibitory control in binge eating disorder relative to other manifestations of obesity. Obesity. (2013) 21:367–77. 10.1002/oby.20068 23404820 PMC3610836

[45] Carbine KA Duraccio KM Kirwan CB Muncy NM LeCheminant JD Larson MJ. A direct comparison between ERP and fMRI measurements of food-related inhibitory control: implications for BMI status and dietary intake. NeuroImage. (2018) 166:335–48. 10.1016/j.neuroimage.2017.11.008 29113942

[46] He Q Xiao L Xue G Wong S Ames SL Schembre SM. Poor ability to resist tempting calorie rich food is linked to altered balance between neural systems involved in urge and self-control. Nutr J. (2014) 13:92. 10.1186/1475-2891-13-92 25228353 PMC4172871

[47] Hege MA Stingl KT Kullmann S Schag K Giel KE Zipfel S. Attentional impulsivity in binge eating disorder modulates response inhibition performance and frontal brain networks. Int J Obes. (2015) 39:353–60. 10.1038/ijo.2014.99 24909828

[48] Hendrick OM Luo X Zhang S Li C-SR. Saliency processing and obesity: a preliminary imaging study of the stop signal task. Obesity. (2012) 20:1796–802. 10.1038/oby.2011.180 21720427 PMC3653271

[49] Hsu J-S Wang P-W Ko C-H Hsieh T-J Chen C-Y Yen J-Y. Altered brain correlates of response inhibition and error processing in females with obesity and sweet food addiction: a functional magnetic imaging study. Obes Res Clin Pract. (2017) 11:677–86. 10.1016/j.orcp.2017.04.011 28552670

[50] Janssen LK Duif I van Loon I Wegman J de Vries JHM Cools R. Loss of lateral prefrontal cortex control in food-directed attention and goal-directed food choice in obesity. NeuroImage. (2017) 146:148–56. 10.1016/j.neuroimage.2016.11.015 27845255

[51] Oliva R Morys F Horstmann A Castiello U Begliomini C. The impulsive brain: neural underpinnings of binge eating behavior in normal-weight adults. Appetite. (2019) 136:33–49. 10.1016/j.appet.2018.12.043 30615922

[52] Scharmüller W Übel S Ebner F Schienle A. Appetite regulation during food cue exposure: a comparison of normal-weight and obese women. Neurosci Lett. (2012) 518:106–10. 10.1016/j.neulet.2012.04.063 22580204

[53] Tuulari JJ Karlsson HK Hirvonen J Salminen P Nuutila P Nummenmaa L. Neural circuits for cognitive appetite control in healthy and obese individuals: an fMRI study. PLoS ONE. (2015) 10:e0116640. 10.1371/journal.pone.0116640 25658479 PMC4320085

[54] Swick D Ashley V Turken U. Are the neural correlates of stopping and not going identical? Quantitative meta-analysis of two response inhibition tasks. NeuroImage. (2011) 56:1655–65. 10.1016/j.neuroimage.2011.02.070 21376819

[55] Banich MT Milham MP Atchley R Cohen NJ Webb A Wszalek T. fMRI studies of stroop tasks reveal unique roles of anterior and posterior brain systems in attentional selection. J Cogn Neurosci. (2000) 12:988–1000. 10.1162/08989290051137521 11177419

[56] Barch DM Braver TS Akbudak E Conturo T Ollinger J Snyder A. Anterior cingulate cortex and response conflict: effects of response modality and processing domain. Cereb Cortex N Y N. (2001) 11:837–48. 10.1093/cercor/11.9.837 11532889

[57] MacDonald AW Cohen JD Stenger VA Carter CS. Dissociating the role of the dorsolateral prefrontal and anterior cingulate cortex in cognitive control. Science. (2000) 288:1835–8. 10.1126/science.288.5472.1835 10846167

[58] Han JE Boachie N Garcia-Garcia I Michaud A Dagher A. Neural correlates of dietary self-control in healthy adults: a meta-analysis of functional brain imaging studies. Physiol Behav. (2018) 192:98–108. 10.1016/j.physbeh.2018.02.037 29496487

[59] Aron AR Poldrack RA. The cognitive neuroscience of response inhibition: relevance for genetic research in attention-deficit/hyperactivity disorder. Biol Psychiatry. (2005) 57:1285–92. 10.1016/j.biopsych.2004.10.026 15950000

[60] Buchsbaum BR Greer S Chang W-L Berman KF. Meta-analysis of neuroimaging studies of the Wisconsin Card-Sorting task and component processes. Hum Brain Mapp. (2005) 25:35–45. 10.1002/hbm.20128 15846821 PMC6871753

[61] Nachev P Kennard C Husain M. Functional role of the supplementary and pre-supplementary motor areas. Nat Rev Neurosci. (2008) 9:856–69. 10.1038/nrn2478 18843271

[62] Duque J Labruna L Verset S Olivier E Ivry RB. Dissociating the role of prefrontal and premotor cortices in controlling inhibitory mechanisms during motor preparation. J Neurosci. (2012) 32:806–16. 10.1523/JNEUROSCI.4299-12.2012 22262879 PMC3304578

[63] Clarke RE Verdejo-Garcia A Andrews ZB. The role of corticostriatal–hypothalamic neural circuits in feeding behaviour: implications for obesity. J Neurochem. (2018) 147:715–29. 10.1111/jnc.14455 29704424

[64] Suzuki S Cross L O'Doherty JP. Elucidating the underlying components of food valuation in the human orbitofrontal cortex. Nat Neurosci. (2017) 20:1780–6. 10.1038/s41593-017-0008-x 29184201 PMC6214455

[65] Hooker CI Knight RT. The role of lateral orbitofrontal cortex in the inhibitory control of emotion. Orbitofrontal Cortex. (2006) 307:307–24. 10.1093/acprof:oso/9780198565741.003.0012

[66] Michaud A Vainik U Garcia-Garcia I Dagher A. Overlapping neural endophenotypes in addiction and obesity. Front Endocrinol. (2017) 8:127. 10.3389/fendo.2017.00127 28659866 PMC5469912

[67] Hollmann M Pleger B Villringer A Horstmann A. Brain imaging in the context of food perception and eating. Curr Opin Lipidol. (2013) 24:18–24. 10.1097/MOL.0b013e32835b61a4 23165087

[68] Rushworth MFS Kennerley SW Walton ME. Cognitive neuroscience: resolving conflict in and over the medial frontal cortex. Curr Biol. (2005) 15:R54–6. 10.1016/j.cub.2004.12.054 15668156

[69] Venkatraman V Payne JW Bettman JR Luce MF Huettel SA. Separate neural mechanisms underlie choices and strategic preferences in risky decision making. Neuron. (2009) 62:593–602. 10.1016/j.neuron.2009.04.007 19477159 PMC3213208

[70] Venkatraman V Rosati AG Taren AA Huettel SA. Resolving response, decision, and strategic control: evidence for a functional topography in dorsomedial prefrontal cortex. J Neurosci. (2009) 29:13158–64. 10.1523/JNEUROSCI.2708-09.2009 19846703 PMC2801415

[71] Bzdok D Langner R Schilbach L Engemann DA Laird AR Fox PT. Segregation of the human medial prefrontal cortex in social cognition. Front Hum Neurosci. (2013) 7:232. 10.3389/fnhum.2013.00232 23755001 PMC3665907

[72] Muhlert N Lawrence AD. Brain structure correlates of emotion-based rash impulsivity. NeuroImage. (2015) 115:138–46. 10.1016/j.neuroimage.2015.04.061 25957991 PMC4463859

[73] Du X Qi X Yang Y Du G Gao P Zhang Y. Corrigendum: altered structural correlates of impulsivity in adolescents with internet gaming disorder. Front Hum Neurosci. (2019) 13:124. 10.3389/fnhum.2019.00124 31024279 PMC6465755

[74] Bechara A Tranel D Damasio H. Characterization of the decision-making deficit of patients with ventromedial prefrontal cortex lesions. Brain. (2000) 123:2189–202. 10.1093/brain/123.11.2189 11050020

[75] Bar-On R Tranel D Denburg NL Bechara A. Exploring the neurological substrate of emotional and social intelligence. Brain. (2003) 126:1790–800. 10.1093/brain/awg177 12805102

[76] de Ruiter MB Oosterlaan J Veltman DJ van den Brink W Goudriaan AE. Similar hyporesponsiveness of the dorsomedial prefrontal cortex in problem gamblers and heavy smokers during an inhibitory control task. Drug Alcohol Depend. (2012) 121:81–9. 10.1016/j.drugalcdep.2011.08.010 21893386

[77] Potenza MN Leung H-C Blumberg HP Peterson BS Fulbright RK Lacadie CM. An fMRI stroop task study of ventromedial prefrontal cortical function in pathological gamblers. Am J Psychiatry. (2003) 160:1990–4. 10.1176/appi.ajp.160.11.1990 14594746

[78] Knoch D Brugger P Regard M. Suppressing versus releasing a habit: frequency-dependent effects of prefrontal transcranial magnetic stimulation. Cereb Cortex. (2005) 15:885–7. 10.1093/cercor/bhh196 15483046

[79] Vanderhasselt M-A De Raedt R Baeken C. Dorsolateral prefrontal cortex and stroop performance: tackling the lateralization. Psychon Bull Rev. (2009) 16:609–12. 10.3758/PBR.16.3.609 19451392

[80] Giuliani NR Mann T Tomiyama AJ Berkman ET. Neural systems underlying the reappraisal of personally craved foods. J Cogn Neurosci. (2014) 26:1390–402. 10.1162/jocn_a_00563 24392892 PMC4081531

[81] Hollmann M Hellrung L Pleger B Schlögl H Kabisch S Stumvoll M. Neural correlates of the volitional regulation of the desire for food. Int J Obes. (2012) 36:648–55. 10.1038/ijo.2011.125 21712804

[82] Lowe CJ Reichelt AC Hall PA. The prefrontal cortex and obesity: a health neuroscience perspective. Trends Cogn Sci. (2019) 23:349–61. 10.1016/j.tics.2019.01.005 30824229

[83] Weygandt M Mai K Dommes E Leupelt V Hackmack K Kahnt T. The role of neural impulse control mechanisms for dietary success in obesity. Neuroimage. (2013) 83:669–78. 10.1016/j.neuroimage.2013.07.028 23867558

[84] Weygandt M Mai K Dommes E Ritter K Leupelt V Spranger J. Impulse control in the dorsolateral prefrontal cortex counteracts post-diet weight regain in obesity. NeuroImage. (2015) 109:318–27. 10.1016/j.neuroimage.2014.12.073 25576647

[85] Li CR Huang C Constable RT Sinha R. Imaging response inhibition in a stop-signal task: neural correlates independent of signal monitoring and post-response processing. J Neurosci. (2006) 26:186–92. 10.1523/JNEUROSCI.3741-05.2006 16399686 PMC6674298

[86] Volz KG Schubotz RI Cramon DY. Variants of uncertainty in decision-making and their neural correlates. Brain Res Bull. (2005) 67:403–12. 10.1016/j.brainresbull.2005.06.011 16216687

[87] Dekleva BM Ramkumar P Wanda PA Kording KP Miller LE. Uncertainty leads to persistent effects on reach representations in dorsal premotor cortex. eLife. (2016) 5:e14316. 10.7554/eLife.14316 27420609 PMC4946902

[88] Goldstein RZ Volkow ND. Dysfunction of the prefrontal cortex in addiction: neuroimaging findings and clinical implications. Nat Rev Neurosci. (2011) 12:652–69. 10.1038/nrn3119 22011681 PMC3462342

[89] Maier SU Hare TA. Higher heart-rate variability is associated with ventromedial prefrontal cortex activity and increased resistance to temptation in dietary self-control challenges. J Neurosci. (2017) 37:446–55. 10.1523/JNEUROSCI.2815-16.2017 28077722 PMC6596577

[90] Levy BJ Wagner AD. Cognitive control and right ventrolateral prefrontal cortex: reflexive reorienting, motor inhibition, and action updating. Ann N Y Acad Sci. (2011) 1224:40–62. 10.1111/j.1749-6632.2011.05958.x 21486295 PMC3079823

[91] Limongi R Pérez FJ. Successful and unsuccessful response inhibitions differentially affect the effective connectivity between insular, presupplementary-motor, and striatal areas. Behav Neurosci. (2017) 131:20–32. 10.1037/bne0000175 28004954

[92] Li C-SR Yan P Chao HH-A Sinha R Paliwal P Constable RT. Error-specific medial cortical and subcortical activity during the stop signal task: a functional magnetic resonance imaging study. Neuroscience. (2008) 155:1142–51. 10.1016/j.neuroscience.2008.06.062 18674592 PMC2605269

[93] Feil J Sheppard D Fitzgerald PB Yücel M Lubman DI Bradshaw JL. Addiction, compulsive drug seeking, and the role of frontostriatal mechanisms in regulating inhibitory control. Neurosci Biobehav Rev. (2010) 35:248–75. 10.1016/j.neubiorev.2010.03.001 20223263

[94] Weafer J Dzemidzic M Eiler W II Oberlin BG Wang Y Kareken DA. Associations between regional brain physiology and trait impulsivity, motor inhibition, and impaired control over drinking. Psychiatry Res Neuroimaging. (2015) 233:81–7. 10.1016/j.pscychresns.2015.04.010 26065376 PMC4536192

[95] Volkow ND Wang GJ Fowler JS Tomasi D Baler R. Food and drug reward: overlapping circuits in human obesity and addiction. In: Carter CS, Dalley JW, editors. Brain Imaging in Behavioral Neuroscience. Berlin: Springer (2012). p. 1–24. 10.1007/7854_2011_169 22016109

[96] Hensler JG. Serotonergic modulation of the limbic system. Neurosci Biobehav Rev. (2006) 30:203–14. 10.1016/j.neubiorev.2005.06.007 16157378

[97] Kötter R Meyer N. The limbic system: a review of its empirical foundation. Behav Brain Res. (1992) 52:105–27. 10.1016/S0166-4328(05)80221-9 1294190

[98] Veldhuizen MG Albrecht J Zelano C Boesveldt S Breslin P Lundström JN. Identification of human gustatory cortex by activation likelihood estimation. Hum Brain Mapp. (2011) 32:2256–66. 10.1002/hbm.21188 21305668 PMC3123671

[99] Craig AD Craig AD. How do you feel–now? The anterior insula and human awareness. Nat Rev Neurosci. (2009) 10:59–70. 10.1038/nrn2555 19096369

[100] Contreras M Ceric F Torrealba F. Inactivation of the interoceptive insula disrupts drug craving and malaise induced by lithium. Science. (2007) 318:655–8. 10.1126/science.1145590 17962567

[101] Naqvi NH Rudrauf D Damasio H Bechara A. Damage to the insula disrupts addiction to cigarette smoking. Science. (2007) 315:531–4. 10.1126/science.1135926 17255515 PMC3698854

[102] Horn NR Dolan M Elliott R Deakin JFW Woodruff PWR. Response inhibition and impulsivity: an fMRI study. Neuropsychologia. (2003) 41:1959–66. 10.1016/S0028-3932(03)00077-0 14572528

[103] Mata F Verdejo-Roman J Soriano-Mas C Verdejo-Garcia A. Insula tuning towards external eating versus interoceptive input in adolescents with overweight and obesity. Appetite. (2015) 93:24–30. 10.1016/j.appet.2015.03.024 25819606

[104] Maddock RJ Garrett AS Buonocore MH. Posterior cingulate cortex activation by emotional words: fMRI evidence from a valence decision task. Hum Brain Mapp. (2003) 18:30–41. 10.1002/hbm.10075 12454910 PMC6871991

[105] Fink GR Markowitsch HJ Reinkemeier M Bruckbauer T Kessler J Heiss W-D. Cerebral representation of one's own past: neural networks involved in autobiographical memory. J Neurosci. (1996) 16:4275–82. 10.1523/JNEUROSCI.16-13-04275.1996 8753888 PMC6579004

[106] Rothemund Y Preuschhof C Bohner G Bauknecht H-C Klingebiel R Flor H. Differential activation of the dorsal striatum by high-calorie visual food stimuli in obese individuals. NeuroImage. (2007) 37:410–21. 10.1016/j.neuroimage.2007.05.008 17566768

[107] Ballmaier M Toga AW Blanton RE Sowell ER Lavretsky H Peterson J. Anterior cingulate, gyrus rectus, and orbitofrontal abnormalities in elderly depressed patients: an MRI-based parcellation of the prefrontal cortex. Am J Psychiatry. (2004) 161:99–108. 10.1176/appi.ajp.161.1.99 14702257

[108] Braver TS Barch DM Gray JR Molfese DL Snyder A. Anterior cingulate cortex and response conflict: effects of frequency, inhibition and errors. Cereb Cortex. (2001) 11:825–36. 10.1093/cercor/11.9.825 11532888

[109] Menon V Adleman NE White CD Glover GH Reiss AL. Error-related brain activation during a Go/NoGo response inhibition task. Hum Brain Mapp. (2001) 12:131–43. 10.1002/1097-0193(200103)12:3&lt;131::AID-HBM1010&gt;3.0.CO;2-C 11170305 PMC6872006

[110] Volkow ND Wang G-J Ma Y Fowler JS Zhu W Maynard L. Expectation enhances the regional brain metabolic and the reinforcing effects of stimulants in cocaine abusers. J Neurosci. (2003) 23:11461–8. 10.1523/JNEUROSCI.23-36-11461.2003 14673011 PMC6740524

[111] Robinson TE Berridge KC. The neural basis of drug craving: An incentive-sensitization theory of addiction. Brain Res Rev. (1993) 18:247–91. 10.1016/0165-0173(93)90013-P 8401595

[112] Millan EZ Ong Z McNally GP. Paraventricular thalamus: gateway to feeding, appetitive motivation, and drug addiction. In: Calvey T, Daniels WMU, editors. Progress in Brain Research. London: Elsevier (2017). p. 113–37. 10.1016/bs.pbr.2017.07.006 29054285

[113] Bhatnagar S Dallman MF. The paraventricular nucleus of the thalamus alters rhythms in core temperature and energy balance in a state-dependent manner. Brain Res. (1999) 851:66–75. 10.1016/S0006-8993(99)02108-3 10642829

[114] Stratford TR Wirtshafter D. Injections of muscimol into the paraventricular thalamic nucleus, but not mediodorsal thalamic nuclei, induce feeding in rats. Brain Res. (2013) 1490:128–33. 10.1016/j.brainres.2012.10.043 23111346 PMC3529785

[115] Zhang X van den Pol AN. Rapid binge-like eating and body weight gain driven by zona incerta GABA neuron activation. Science. (2017). 356:853–9. 10.1126/science.aam7100 28546212 PMC6602535

[116] Balleine BW Delgado MR Hikosaka O. The role of the dorsal striatum in reward and decision-making. J Neurosci. (2007) 27:8161–5. 10.1523/JNEUROSCI.1554-07.2007 17670959 PMC6673072

[117] Volkow ND Wang G-J Fowler JS Logan J Jayne M Franceschi D. “Nonhedonic” food motivation in humans involves dopamine in the dorsal striatum and methylphenidate amplifies this effect. Synapse. (2002) 44:175–80. 10.1002/syn.10075 11954049

[118] Green E Jacobson A Haase L Murphy C. Reduced nucleus accumbens and caudate nucleus activation to a pleasant taste is associated with obesity in older adults. Brain Res. (2011) 1386:109–17. 10.1016/j.brainres.2011.02.071 21362414 PMC3086067

[119] Babbs RK Sun X Felsted J Chouinard-Decorte F Veldhuizen MG Small DM. Decreased caudate response to milkshake is associated with higher body mass index and greater impulsivity. Physiol Behav. (2013) 121:103–11. 10.1016/j.physbeh.2013.03.025 23562867 PMC3731396

[120] Buchwald NA Wyers EJ Lauprecht CW Heuser G. The “caudate-spindle” IV. A behavioral index of caudate-induced inhibition. Electroencephalogr Clin Neurophysiol. (1961) 13:531–7. 10.1016/0013-4694(61)90167-5 13874289

[121] Kitsikis A Rougeul A. The effect of caudate stimulation on conditioned motor behavior in monkeys. Physiol Behav. (1968) 3:831–7. 10.1016/0031-9384(68)90163-717258302

[122] Aron AR Schlaghecken F Fletcher PC Bullmore ET Eimer M Barker R. Inhibition of subliminally primed responses is mediated by the caudate and thalamus: evidence from functional MRI and Huntington's disease. Brain. (2003) 126:713–23. 10.1093/brain/awg067 12566291 PMC3838934

[123] Martikainen IK Nuechterlein EB Peciña M Love TM Cummiford CM Green CR. Chronic back pain is associated with alterations in dopamine neurotransmission in the ventral striatum. J Neurosci. (2015) 35:9957–65. 10.1523/JNEUROSCI.4605-14.2015 26156996 PMC4495244

[124] Postuma RB Dagher A. Basal ganglia functional connectivity based on a meta-analysis of 126 positron emission tomography and functional magnetic resonance imaging publications. Cereb Cortex. (2006) 16:1508–21. 10.1093/cercor/bhj088 16373457

[125] Leh SE Ptito A Chakravarty MM Strafella AP. Fronto-striatal connections in the human brain: a probabilistic diffusion tractography study. Neurosci Lett. (2007) 419:113–8. 10.1016/j.neulet.2007.04.049 17485168 PMC5114128

[126] Di Martino A Scheres A Margulies DS Kelly AMC Uddin LQ Shehzad Z. Functional connectivity of human striatum: a resting state fMRI study. Cereb Cortex. (2008) 18:2735–47. 10.1093/cercor/bhn041 18400794

[127] Choi EY Yeo BTT Buckner RL. The organization of the human striatum estimated by intrinsic functional connectivity. J Neurophysiol. (2012) 108:2242–63. 10.1152/jn.00270.2012 22832566 PMC3545026

[128] Jung WH Jang JH Park JW Kim E Goo E-H Im O-S. Unravelling the intrinsic functional organization of the human striatum: a parcellation and connectivity study based on resting-state fMRI. PLoS ONE. (2014) 9:e106768. 10.1371/journal.pone.0106768 25203441 PMC4159235

[129] Bragulat V Dzemidzic M Bruno C Cox CA Talavage T Considine RV. Food-related odor probes of brain reward circuits during hunger: a pilot fMRI study. Obesity. (2010) 18:1566–71. 10.1038/oby.2010.57 20339365

[130] St-Onge M-P Sy M Heymsfield SB Hirsch J. Human cortical specialization for food: a functional magnetic resonance imaging investigation. J Nutr. (2005) 135:1014–8. 10.1093/jn/135.5.1014 15867274

[131] Yokum S Gearhardt AN Harris JL Brownell KD Stice E. Individual differences in striatum activity to food commercials predict weight gain in adolescents. Obesity. (2014) 22:2544–51. 10.1002/oby.20882 25155745 PMC4236252

[132] Berthoud H-R. Multiple neural systems controlling food intake and body weight. Neurosci Biobehav Rev. (2002) 26:393–428. 10.1016/S0149-7634(02)00014-3 12204189

[133] Chen S Dong D Jackson T Zhuang Q Chen H. Trait-based food-cravings are encoded by regional homogeneity in the parahippocampal gyrus. Appetite. (2017) 114:155–60. 10.1016/j.appet.2017.03.033 28344152

[134] Nakata H Sakamoto K Ferretti A Gianni Perrucci M Del Gratta C Kakigi R. Somato-motor inhibitory processing in humans: an event-related functional MRI study. NeuroImage. (2008) 39:1858–66. 10.1016/j.neuroimage.2007.10.041 18083602

[135] Schmidt A Müller F Lenz C Dolder PC Schmid Y Zanchi D. Acute LSD effects on response inhibition neural networks. Psychol Med. (2018) 48:1464–73. 10.1017/S0033291717002914 28967351

[136] Wodka EL Mahone EM Blankner JG Larson JCG Fotedar S Denckla MB. Evidence that response inhibition is a primary deficit in ADHD. J Clin Exp Neuropsychol. (2007) 29:345–56. 10.1080/13803390600678046 17497558

[137] Spinelli S Joel S Nelson TE Vasa RA Pekar JJ Mostofsky SH. Different neural patterns are associated with trials preceding inhibitory errors in children with and without attention-deficit/hyperactivity disorder. J Am Acad Child Adolesc Psychiatry. (2011) 50:705–15.e3. 10.1016/j.jaac.2011.03.014 21703498 PMC3971481

[138] Sheinkopf SJ Lester BM Sanes JN Eliassen JC Hutchison ER Seifer R. Functional MRI and response inhibition in children exposed to cocaine *in utero*. Dev Neurosci. (2009) 31:159–66. 10.1159/000207503 19372696 PMC2951722

[139] Cohen RA. Cuneus. In: Kreutzer JS, DeLuca J, Caplan B, editors. Encyclopedia of Clinical Neuropsychology. New York, NY: Springer (2011). p. 756–7.

[140] Connolly CG Bell RP Foxe JJ Garavan H. Dissociated grey matter changes with prolonged addiction and extended abstinence in cocaine users. PLoS ONE. (2013) 8:e59645. 10.1371/journal.pone.0059645 23527239 PMC3601087

[141] Wang J Fan Y Dong Y Ma M Dong Y Niu Y. Combining gray matter volume in the cuneus and the cuneus-prefrontal connectivity may predict early relapse in abstinent alcohol-dependent patients. PLoS ONE. (2018) 13:e0196860. 10.1371/journal.pone.0196860 29734343 PMC5937790

[142] Goldstein RZ Tomasi D Rajaram S Cottone LA Zhang L Maloney T. Role of the anterior cingulate and medial orbitofrontal cortex in processing drug cues in cocaine addiction. Neuroscience. (2007) 144:1153–9. 10.1016/j.neuroscience.2006.11.024 17197102 PMC1852512

[143] Ng J Stice E Yokum S Bohon C. An fMRI study of obesity, food reward, and perceived caloric density. Does a low-fat label make food less appealing? Appetite. (2011) 57:65–72. 10.1016/j.appet.2011.03.017 21497628 PMC3124617

[144] Stice E Spoor S Bohon C Veldhuizen MG Small DM. Relation of reward from food intake and anticipated food intake to obesity: a functional magnetic resonance imaging study. J Abnorm Psychol. (2008) 117:924–35. 10.1037/a0013600 19025237 PMC2681092

[145] Hill SY Sharma V Jones BL. Lifetime use of cannabis from longitudinal assessments, cannabinoid receptor (CNR1) variation, and reduced volume of the right anterior cingulate. Psychiatry Res Neuroimaging. (2016) 255:24–34. 10.1016/j.pscychresns.2016.05.009 27500453 PMC5025865

[146] Hall W Carter A Forlini C. The brain disease model of addiction: is it supported by the evidence and has it delivered on its promises? Lancet Psychiatry. (2015) 2:105–10. 10.1016/S2215-0366(14)00126-6 26359616

[147] Willeumier K Taylor DV Amen DG. Decreased cerebral blood flow in the limbic and prefrontal cortex using SPECT imaging in a cohort of completed suicides. Transl Psychiatry. (2011) 1:e28. 10.1038/tp.2011.28 22832602 PMC3309501

[148] Klonsky ED May A. Rethinking impulsivity in suicide. Suicide Life Threat Behav. (2010) 40:612–9. 10.1521/suli.2010.40.6.612 21198330

[149] Steele VR Aharoni E Munro GE Calhoun VD Nyalakanti P Stevens MC. A large scale (*N* = 102) functional neuroimaging study of response inhibition in a Go/NoGo task. Behav Brain Res. (2013) 256:529–36. 10.1016/j.bbr.2013.06.001 23756137 PMC4437665

[150] Farr OM Hu S Zhang S Li CR. Decreased saliency processing as a neural measure of Barratt impulsivity in healthy adults. NeuroImage. (2012) 63:1070–7. 10.1016/j.neuroimage.2012.07.049 22885245 PMC3472158

[151] Garavan H Ross TJ Stein EA. Right hemispheric dominance of inhibitory control: an event-related functional MRI study. Proc Natl Acad Sci. (1999) 96:8301–6. 10.1073/pnas.96.14.8301 10393989 PMC22229

[152] Hedden T Gabrieli JDE. Shared and selective neural correlates of inhibition, facilitation, and shifting processes during executive control. NeuroImage. (2010) 51:421–31. 10.1016/j.neuroimage.2010.01.089 20123030 PMC2852172

[153] Gröpper S Spengler S Stuke H Gawron CK Parnack J Gutwinski S. Behavioral impulsivity mediates the relationship between decreased frontal gray matter volume and harmful alcohol drinking: a voxel-based morphometry study. J Psychiatr Res. (2016) 83:16–23. 10.1016/j.jpsychires.2016.08.006 27529648

[154] Carmona S Vilarroya O Bielsa A Trèmols V Soliva JC Rovira M. Global and regional gray matter reductions in ADHD: a voxel-based morphometric study. Neurosci Lett. (2005) 389:88–93. 10.1016/j.neulet.2005.07.020 16129560

[155] Schilling C Kühn S Romanowski A Schubert F Kathmann N Gallinat J. Cortical thickness correlates with impulsiveness in healthy adults. NeuroImage. (2012) 59:824–30. 10.1016/j.neuroimage.2011.07.058 21827861

[156] Stoeckel LE Murdaugh DL Cox JE Cook EW Weller RE. Greater impulsivity is associated with decreased brain activation in obese women during a delay discounting task. Brain Imaging Behav. (2013) 7:116–28. 10.1007/s11682-012-9201-4 22948956 PMC3561478

[157] Arias-Carrión O Stamelou M Murillo-Rodríguez E Menéndez-González M Pöppel E. Dopaminergic reward system: a short integrative review. Int Arch Med. (2010) 3:24. 10.1186/1755-7682-3-24 20925949 PMC2958859

[158] Schultz W. Multiple reward signals in the brain. Nat Rev Neurosci. (2000) 1:199–207. 10.1038/35044563 11257908

[159] Wickens JR Horvitz JC Costa RM Killcross S. Dopaminergic mechanisms in actions and habits. J Neurosci. (2007) 27:8181–3. 10.1523/JNEUROSCI.1671-07.2007 17670964 PMC6673057

[160] Nelson A Killcross S. Amphetamine exposure enhances habit formation. J Neurosci. (2006) 26:3805–12. 10.1523/JNEUROSCI.4305-05.2006 16597734 PMC6674135

[161] Johnson AW. Eating beyond metabolic need: how environmental cues influence feeding behavior. Trends Neurosci. (2013) 36:101–9. 10.1016/j.tins.2013.01.002 23333343

[162] Luijten M Veltman DJ van den Brink W Hester R Field M Smits M. Neurobiological substrate of smoking-related attentional bias. NeuroImage. (2011) 54:2374–81. 10.1016/j.neuroimage.2010.09.064 20932921

[163] Crick FC Koch C. What is the function of the claustrum? Philos Trans R Soc B Biol Sci. (2005) 360:1271–9. 10.1098/rstb.2005.1661 16147522 PMC1569501

[164] Torgerson CM Irimia A Goh SYM Horn JDV. The DTI connectivity of the human claustrum. Hum Brain Mapp. (2015) 36:827–38. 10.1002/hbm.22667 25339630 PMC4324054

[165] Mathur BN. The claustrum in review. Front Syst Neurosci. (2014) 8:48. 10.3389/fnsys.2014.00048 24772070 PMC3983483

[166] Goll Y Atlan G Citri A. Attention: the claustrum. Trends Neurosci. (2015) 38:486–95. 10.1016/j.tins.2015.05.006 26116988

[167] Wang G-J Volkow ND Logan J Pappas NR Wong CT Zhu W. Brain dopamine and obesity. Lancet. (2001) 357:354–7. 10.1016/S0140-6736(00)03643-6 11210998

[168] Josipovic Z. Nondual awareness: consciousness-as-such as non-representational reflexivity. In: Srinivasan N, editor. Progress in Brain Research. London: Elsevier (2019). p. 273–98. 10.1016/bs.pbr.2018.10.021 30732841

[169] van der Laan LN de Ridder DTD Viergever MA Smeets PAM. The first taste is always with the eyes: a meta-analysis on the neural correlates of processing visual food cues. NeuroImage. (2011) 55:296–303. 10.1016/j.neuroimage.2010.11.055 21111829

[170] American Psychiatric Association. Diagnostic and Statistical Manual for Mental Disorders, 5th Edition: DSM-5. Washington, DC: American Psychiatric Publishing (2013). 10.1176/appi.books.9780890425596

[171] Hudson JI Hiripi E Pope HG Kessler RC. The prevalence and correlates of eating disorders in the national comorbidity survey replication. Biol Psychiatry. (2007) 61:348–58. 10.1016/j.biopsych.2006.03.040 16815322 PMC1892232

[172] Wilfley DE Friedman MA Dounchis JZ Stein RI Welch RR Ball SA. Comorbid psychopathology in binge eating disorder: relation to eating disorder severity at baseline and following treatment. J Consult Clin Psychol. (2000) 68:641. 10.1037/0022-006X.68.4.641 10965639

[173] Yanovski SZ Nelson JE Dubbert BK Spitzer RL. Association of binge eating disorder and psychiatric comorbidity in obese subjects. Am J Psychiatry. (1993) 150:1472–9. 10.1176/ajp.150.10.1472 8379549

[174] Johnson JG Spitzer RL Williams JBW. Health problems, impairment and illnesses associated with bulimia nervosa and binge eating disorder among primary care and obstetric gynaecology patients. Psychol Med. (2001) 31:1455–66. 10.1017/S0033291701004640 11722160

[175] Bulik CM Sullivan PF Kendler KS. Medical and psychiatric morbidity in obese women with and without binge eating. Int J Eat Disord. (2002) 32:72–8. 10.1002/eat.10072 12183948

[176] Mobbs O Iglesias K Golay A Van der Linden M. Cognitive deficits in obese persons with and without binge eating disorder. Investigation using a mental flexibility task. Appetite. (2011) 57:263–71. 10.1016/j.appet.2011.04.023 21600255

[177] Fernández-Aranda F Pinheiro AP Thornton LM Berrettini WH Crow S Fichter MM. Impulse control disorders in women with eating disorders. Psychiatry Res. (2008) 157:147–57. 10.1016/j.psychres.2007.02.011 17961717

[178] Bočková M Chládek J Jurák P Halámek J Rektor I. Executive functions processed in the frontal and lateral temporal cortices: intracerebral study. Clin Neurophysiol. (2007) 118:2625–36. 10.1016/j.clinph.2007.07.025 17911041

[179] Ojemann GA Schoenfield-McNeill J. Activity of neurons in human temporal cortex during identification and memory for names and words. J Neurosci. (1999) 19:5674–82. 10.1523/JNEUROSCI.19-13-05674.1999 10377373 PMC6782319

[180] Claus ED Kiehl KA Hutchison KE. Neural and behavioral mechanisms of impulsive choice in alcohol use disorder. Alcohol Clin Exp Res. (2011) 35:1209–19. 10.1111/j.1530-0277.2011.01455.x 21676001 PMC3117198

[181] Goswami R Dufort P Tartaglia MC Green RE Crawley A Tator CH. Frontotemporal correlates of impulsivity and machine learning in retired professional athletes with a history of multiple concussions. Brain Struct Funct. (2016) 221:1911–25. 10.1007/s00429-015-1012-0 25721800 PMC4853456

[182] Soloff P Nutche J Goradia D Diwadkar V. Structural brain abnormalities in borderline personality disorder: a voxel-based morphometry study. Psychiatry Res Neuroimaging. (2008) 164:223–36. 10.1016/j.pscychresns.2008.02.003 19019636 PMC3286221

[183] Goethals I Audenaert K Jacobs F den Eynde FV Bernagie K Kolindou A. Brain perfusion SPECT in impulsivity-related personality disorders. Behav Brain Res. (2005) 157:187–92. 10.1016/j.bbr.2004.06.022 15617785

[184] King BM Kass JM Cadieux NL Sam H Neville KL Arceneaux ER. Hyperphagia and obesity in female rats with temporal lobe lesions. Physiol Behav. (1993) 54:759–65. 10.1016/0031-9384(93)90088-W 8248354

[185] Kurth F Levitt JG Phillips OR Luders E Woods RP Mazziotta JC. Relationships between gray matter, body mass index, and waist circumference in healthy adults. Hum Brain Mapp. (2013) 34:1737–46. 10.1002/hbm.22021 22419507 PMC6869996

[186] Taki Y Kinomura S Sato K Inoue K Goto R Okada K. Relationship between body mass index and gray matter volume in 1,428 healthy individuals. Obesity. (2008) 16:119–24. 10.1038/oby.2007.4 18223623

[187] Raji CA Ho AJ Parikshak NN Becker JT Lopez OL Kuller LH. Brain structure and obesity. Hum Brain Mapp. (2010) 31:353–64. 10.1002/hbm.20870 19662657 PMC2826530

[188] Jagust W. What Can Imaging Reveal about Obesity and the Brain? Curr Alzheimer Res. (2007) 4:135–9. 10.2174/156720507780362146 17430236

[189] Zhang Y Ji G Xu M Cai W Zhu Q Qian L. Recovery of brain structural abnormalities in morbidly obese patients after bariatric surgery. Int J Obes. (2016) 40:1558–65. 10.1038/ijo.2016.98 27200505

[190] Gustafson D Lissner L Bengtsson C Björkelund C Skoog I. A 24-year follow-up of body mass index and cerebral atrophy. Neurology. (2004) 63:1876–81. 10.1212/01.WNL.0000141850.47773.5F 15557505

[191] Lowe MR van Steenburgh J Ochner C Coletta M. Neural correlates of individual differences related to appetite. Physiol Behav. (2009) 97:561–71. 10.1016/j.physbeh.2009.04.001 19361535

[192] Davis C. From passive overeating to “food addiction”: a spectrum of compulsion and severity. ISRN Obes. (2013) 2013:435027. 10.1155/2013/435027 24555143 PMC3901973

[193] Fuentes P Barrós-Loscertales A Bustamante JC Rosell P Costumero V Ávila C. Individual differences in the behavioral inhibition system are associated with orbitofrontal cortex and precuneus gray matter volume. Cogn Affect Behav Neurosci. (2012) 12:491–8. 10.3758/s13415-012-0099-5 22592859

[194] Ikeda A Ohara S Matsumoto R Kunieda T Nagamine T Miyamoto S. Role of primary sensorimotor cortices in generating inhibitory motor response in humans. Brain J Neurol. (2000) 123:1710–21. 10.1093/brain/123.8.1710 10908200

[195] Peterburs J Desmond JE. The role of the human cerebellum in performance monitoring. Curr Opin Neurobiol. (2016) 40:38–44. 10.1016/j.conb.2016.06.011 27372055 PMC5056810

